# Supplementary material for: Quality of maternal healthcare and travel time influence birthing service utilisation in Ghanaian health facilities: a geographical analysis of routine health data
Source: BMJ Open. 2023 Jan 18;13(1):e066792. doi: 10.1136/bmjopen-2022-066792 (PMC9853258; doi:10.1136/bmjopen-2022-066792)

Supplementary material

Supplemental appendix 1: Comparison between demand aggregated at sub-district versus travel time to place of residence cost allocation

Model	AIC	AIC Change
Travel time + summary quality index + number of women 15 to 45 years near the catchment of a town (origin) estimated by travel time to place of residence cost allocation.	84,796.6	0
Travel time + summary quality index + number of women 15 to 45 years in origin sub-district	92,298.1	7501.47

Supplemental appendix 2: Travel speed assigned to land cover and road types

Feature	Travel speeds (Kmh^{-1})	
	Multi modal	Walking
Land cover		
Grassland and built-up areas	5	5
Trees, shrubland, cropland, bare or sparse vegetation	4	4
Herbaceous wetland	3	3
Rivers and lakes	0	0
Roads		
Trunk	48	5
Primary	43	5
Secondary	40	5
Tertiary	35	5
Tracks and residential	30	5
Footpaths	5	5

Supplemental appendix 3: Proportionate weighted estimation of mechanised travel speeds by rood smoothness and road class

Road smoothness description by OSM	Average speed by road smoothness (A)	% primary (B)	A*B	% secondary (C)	A*C	% tertiary (D)	A*D	% track (E)	A*E	% trunk (F)	A*F	% All roads (G)	A*G
Horrible	20	0	0.0	2	0.3	1	0.2	9	1.8	0	0.0	4	0.7
Very bad	31	14	4.2	16	4.8	58	18.0	80	24.9	1	0.3	45	14.0
bad	31	16	5.1	30	9.2	19	5.9	9	2.9	1	0.3	14	4.2
good	48	65	31.0	49	23.6	16	7.6	1	0.4	98	46.9	35	16.7
intermediate	48	5	2.6	4	1.9	5	2.4	0	0.1	0	0.0	2	1.2
excellent	48	0	0.0	0	0.0	1	0.4	0	0.1	0	0.0	0	0.1
Speed applied to road types		100	43	100	40	100	35	100	30	100	48	100	37

The OSM road smoothness are described here: **https://wiki.openstreetmap.org/wiki/Key:smoothness**

Supplemental appendix 4:Relative performance of quality care indices in predicting the count of women giving birth between town-health facilities

Model	AIC	AIC Change
Travel time + number of women 15 to 45 years in origin sub-district + summary quality index (human resource capacity, signal functions, medicines, logistics, amenities, referral capacity, motivation, privacy, training and wash)	84,796.65	0
Travel time + number of women 15 to 45 years in origin sub-district + summary quality index quintiles	84,885.28	88.54
Travel time + number of women 15 to 45 years in origin sub-district + size of health facility (human resource capacity, signal functions, medicines, logistics, amenities, referral capacity)	84,328.42	502.76
Travel time + number of women 15 to 45 years in origin sub-district +health facility type (CHPS, health center, hospital, polyclinic, maternity home)	85,407.18	610.53
Travel time + number of women 15 to 45 years in origin sub-district + Number of EmONC signal functions	85,953.44	1156.79
Travel time + number of women 15 to 45 years in origin sub-district +health facility level (primary or secondary)	86,078.33	1281.68
Travel time + number of women 15 to 45 years in origin sub-district + routine quality (staff motivation, privacy, training, wash)	89,608.96	5478.0.03

Supplemental appendix 5: Breakdown of data availability and missing data by health facility ownership

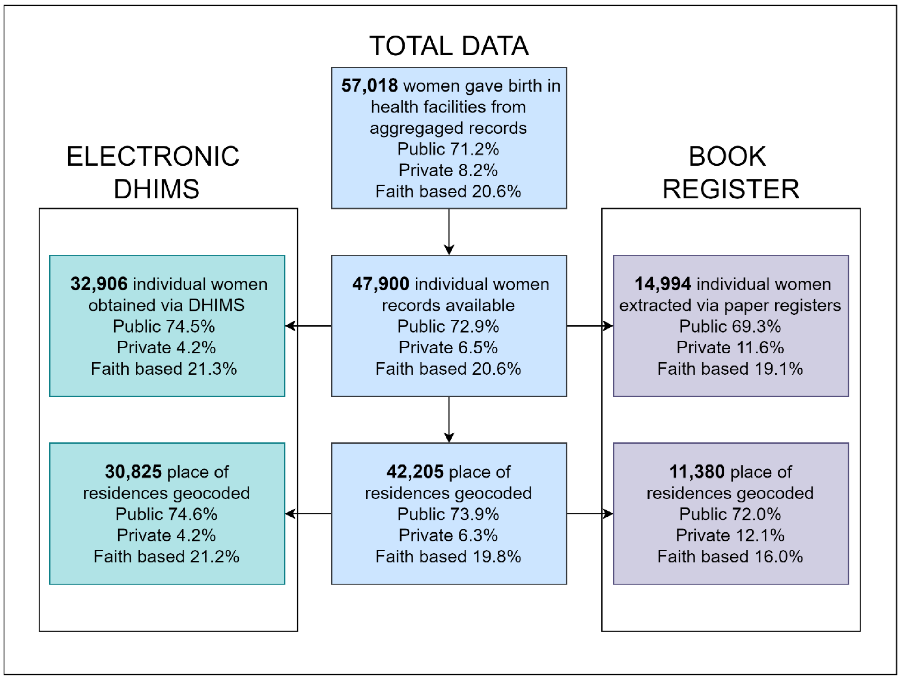

Supplemental appendix 6: Correlation between (A) Aggregated versus individual data, (B) Individual data versus geocoded individual data, and (C) Aggregated versus individual geocoded data

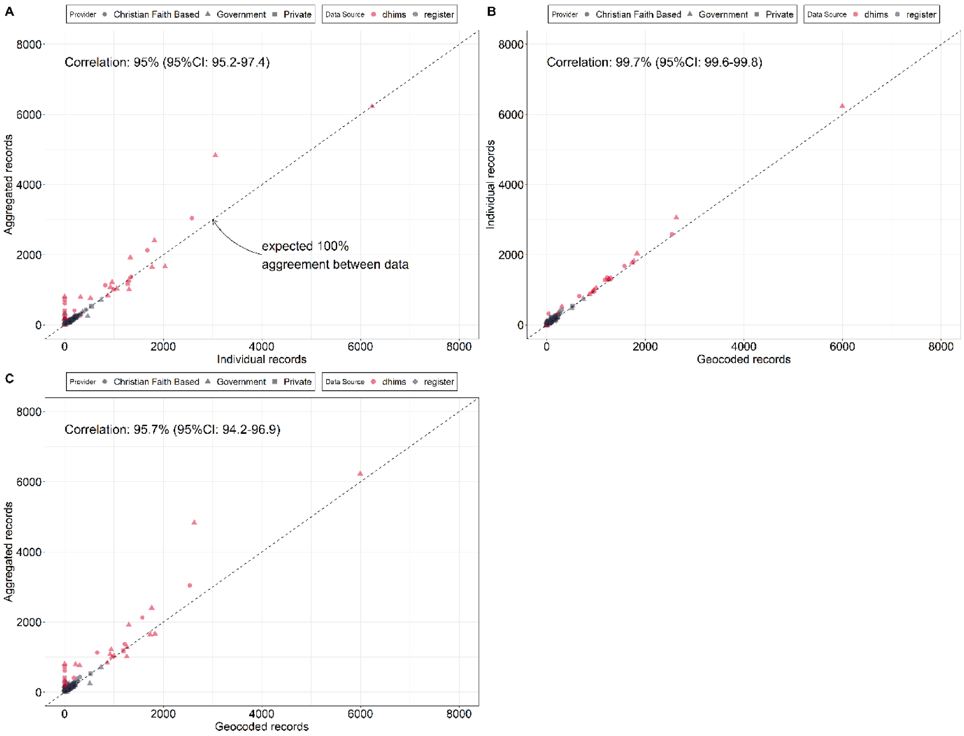

Supplement: Supplementary data [file bmjopen-2022-066792supp001.pdf]
