# Supplementary material for: Quality of maternal healthcare and travel time influence birthing service utilisation in Ghanaian health facilities: a geographical analysis of routine health data
Source: BMJ Open. 2023 Jan 18;13(1):e066792. doi: 10.1136/bmjopen-2022-066792 (PMC9853258; doi:10.1136/bmjopen-2022-066792)
Supplement: Author's manuscript [file bmjopen-2022-066792.draft_revisions.pdf]

BMJ Open

BMJ Open is committed to open peer review. As part of this commitment we make the peer review history of every article we publish publicly available.

When an article is published we post the peer reviewers' comments and the authors' responses online. We also post the versions of the paper that were used during peer review. These are the versions that the peer review comments apply to.

The versions of the paper that follow are the versions that were submitted during the peer review process. They are not the versions of record or the final published versions. They should not be cited or distributed as the published version of this manuscript.

BMJ Open is an open access journal and the full, final, typeset and author-corrected version of record of the manuscript is available on our site with no access controls, subscription charges or pay-per-view fees (http://bmjopen.bmj.com).

If you have any questions on BMJ Open's open peer review process please email info.bmjopen@bmj.com

Quality of maternal health care and travel time influence birthing service utilisation in Ghanaian health facilities: A spatial interaction model

Journal:	*BMJ Open*
Manuscript ID	bmjopen-2022-066792
Article Type:	Original research
Date Submitted by the Author:	19-Jul-2022
Complete List of Authors:	Dotse-Gborgbortsi, Winfred; University of Southampton, School of Geography and Environmental Science; WorldPop, School of Geography and Environmental Science, University of Southampton<br>Tatem, Andrew; University of Southampton, School of Geography and Environmental Science; WorldPop, School of Geography and Environmental Science, University of Southampton<br>Matthews, Zoe; University of Southampton, Department of Social Statistics and Demography<br>Alegana, V; Kenya Medical Research Institute, Population Health Unit - Wellcome Trust Research Programme<br>Ofosu, Anthony; Ghana Health Service, Headquarters<br>Wright, Jim; University of Southampton, School of Geography and Environmental Science
Keywords:	EPIDEMIOLOGY, Maternal medicine < OBSTETRICS, Health policy < HEALTH SERVICES ADMINISTRATION & MANAGEMENT, PUBLIC HEALTH

SCHOLARONE™
Manuscripts

I, the Submitting Author has the right to grant and does grant on behalf of all authors of the Work (as defined in the below author licence), an exclusive licence and/or a non-exclusive licence for contributions from authors who are: i) UK Crown employees; ii) where BMJ has agreed a CC-BY licence shall apply, and/or iii) in accordance with the terms applicable for US Federal Government officers or employees acting as part of their official duties; on a worldwide, perpetual, irrevocable, royalty-free basis to BMJ Publishing Group Ltd ("BMJ") its licensees and where the relevant Journal is co-owned by BMJ to the co-owners of the Journal, to publish the Work in this journal and any other BMJ products and to exploit all rights, as set out in our licence.

The Submitting Author accepts and understands that any supply made under these terms is made by BMJ to the Submitting Author unless you are acting as an employee on behalf of your employer or a postgraduate student of an affiliated institution which is paying any applicable article publishing charge ("APC") for Open Access articles. Where the Submitting Author wishes to make the Work available on an Open Access basis (and intends to pay the relevant APC), the terms of reuse of such Open Access shall be governed by a Creative Commons licence – details of these licences and which Creative Commons licence will apply to this Work are set out in our licence referred to above.

Other than as permitted in any relevant BMJ Author's Self Archiving Policies, I confirm this Work has not been accepted for publication elsewhere, is not being considered for publication elsewhere and does not duplicate material already published. I confirm all authors consent to publication of this Work and authorise the granting of this licence.

For peer review only

Quality of maternal health care and travel time influence birthing service utilisation in Ghanaian health facilities: A spatial interaction model

Winfred Dotse-Gborgbortsi [1,2], Andrew J Tatem [1,2], Zoë Matthews [3], Victor Alegana [4], Anthony Ofosu [5], Jim Wright [1].

[1] School of Geography and Environmental Science, University of Southampton, Southampton, UK

[2] WorldPop, School of Geography and Environmental Science, University of Southampton, Southampton, Southampton, UK

[3] Department of Social Statistics and Demography, University of Southampton, Southampton, UK

[4] Population Health Unit, Kenya Medical Research Institute – Wellcome Trust Research Programme, Nairobi, Kenya

[5] Ghana Health Service, Headquarters, Accra, Ghana

Corresponding author: Winfred Dotse-Gborgbortsi, w.w.dotse-gborgbortsi@soton.ac.uk

For peer review only

Abstract

Introduction Quality of maternal health services and travels both affect maternal health service utilisation, but few studies in Sub-Saharan Africa have assessed how service quality and proximity interact to affect utilisation. This study used geographic accessibility analysis to investigate how quality maternal health services and travel times interact to affect birthing service utilisation in Eastern Region, Ghana.

Methods This cross-sectional study uses a spatial interaction model to predict utilisation rates via a zero-inflated negative binomial spatial interaction gravity model. Through spatial linkage, the model integrated routinely collected birth records from health facilities, health facility service provision assessments, population estimates and travel time. The birth records were from January to December 2017 obtained from the Ghana Health Service District Health Information Management Systems or manually extracted from hand written registers.

Results As travel time from women's place of residence to the health facility increased up to two hours, the utilisation rate markedly decreased but higher quality of maternal health services have a larger, positive effect on utilisation rates than service proximity. The quality of maternal health services was higher in hospitals than in primary care facilities. Travelling via mechanised transport, 88.6% of women 15 to 45 years were within two hours of any birthing service. For critical comprehensive emergency obstetric care (CEmONC) services, the majority (56.2%) of women were beyond the two-hour threshold. The few CEmONC services were in urban centres, disadvantaging rural populations.

Conclusion To increase birthing service utilisation in Ghana, higher quality health facilities should be sited closer to women, particularly in rural areas. Beyond Ghana, routinely collected birth records could be used to understand the interaction of service proximity and quality.

Keywords: Geographic accessibility, maternal health, Health Information Systems, Geographic Information Systems, Health Services Accessibility,

For peer review only

Strengths and limitations of this study

Through spatial integration of routine health data, quality care metrics, and population data, this study reveals how healthcare quality and geographic access to care interact to affect birthing service utilisation.

Most studies of birthing service access and utilisation use household survey data with approximate residential locations for women and without details of specific health facilities attended but we used routine health data containing such information.

This study demonstrates the utility of routine health data for maternal health research and interventions, especially in settings with digitised individual routine health data, service quality information and population data.

Women whose residential address could not be located were excluded from the study but this did not lead to a selection bias.

The model requires aggregated data so the study could not account for individual socio-demographic characteristics that affect the utilisation of birthing services.

For peer review only

Introduction

Gaps in maternal health system quality in low and middle income countries (LMICs) have led to excess obstetric complications and maternal deaths [1]. Though quality maternal health care during labour is vital in reducing maternal mortality [2], and despite the steady rise in skilled birth coverage, maternity services remain below acceptable levels in most of sub-Saharan Africa [3]. The rising rate of health facility births is not associated with a decline in maternal deaths due to substandard health facilities incapable of averting maternal deaths [4]. Indeed, an estimated 92.5% of Sub-Saharan Africa's population is within two hours' journey time of a hospital [5] but maternal mortality is indicative of poor care.

There is ample evidence on the determinants of health facility utilisation for birth and the causes of maternal mortality [6 7]. Increasing distance to the closest health facility decreases the odds of health facility births [8] and increases maternal mortality rates [9]. For referred cases between health facilities too, the maternal health risk is higher for longer travel times [10]. Geographic accessibility hinders decisions to attend a health facility during pregnancy or seek care in a health facility for complications during home birth [11]. Furthermore, after deciding to use a health facility, proximity to care influences the choice of facility, journey time and transportation mode [12]. Therefore, to prevent maternal deaths, it is recommended that quality health facilities be within two hours' journey time of residential locations [13].

Research across LMICs rarely uses routine health data to evaluate the relationship between quality maternal health care and birthing service utilisation because they can be incomplete, difficult to access, and expensive to extract and process [14 15]. Most studies conduct one-off sub-national surveys to collect data [16 17] or rely on national household surveys such as the Demographic and Health Survey [18 19]. However, surveys can be expensive and infrequent. Despite the challenges with routine health data, it has become increasingly important for malaria and maternal health research in Sub-Saharan Africa in recent years [20].

The quality of maternal health care impacts the decision to seek care and timely, adequate care when women reach a health facility. A review of supply-side barriers limiting access to quality maternal health care found drugs and equipment, staff capacity and morale, infrastructure and referral systems as some of the challenges [21]. In

For peer review only

addition, privacy during consultation and labour, staff morale, cleanliness, and well-kept physical surroundings were other factors that determined service satisfaction among women seeking birthing services [22]. Thus, women are likely to travel longer to receive care in a health facility perceived as better than the nearest one [23].

There is inadequate evidence on healthcare utilisation and proximity to quality maternal care to support Ghana's maternal health interventions and programming. The latest analysis calculating journey times to different qualities of maternal health services is over a decade old [24]. Furthermore, the study measures potential access and did not estimate the effect of quality on utilisation rates. Two recent studies integrated quality care indicators estimated via service provision assessment (SPA) with secondary data analysis to predict the probability of skilled attendance at birth [25] and health facility births [26]. However, both studies estimated journeys to the nearest health facility, assuming women do not bypass health facilities to seek care elsewhere [27]. In the absence of detailed spatial information on where women reside and the actual health facilities they use, realised access modelling is challenging.

Building on previous work [27], this study firstly aims to determine the effect of quality maternal health services and travel time on birthing service utilisation via geographic accessibility analysis by integrating routine birth data, SPA, and ancillary spatial data. Utilisation is modelled as a product of travel time from place of residence to health facilities, population demand in place of residence and maternal service quality in health facilities. Secondly, the study aims to assess how different facility characteristics influence utilisation. Finally, it aims to estimate the number of women within 2 hours' walk or mechanised journey from any birthing service and from comprehensive emergency obstetric and newborn care services (CEmONC).

Methods

Research design and setting

The study is a cross-sectional spatial interaction analysis of birth service utilisation. The gravity models in this study predict the number of women making trips from a place of residence to a health facility to give birth as a function of travel times, the population of women, and the quality of maternal health services.

For peer review only

The study was conducted in Eastern Region, Ghana. The Eastern Region has 33 administrative districts divided into 225 sub-districts. The 2021 population census estimates 2.9 million persons in the region of which approximately 50.8% are females and 51.5% live in rural areas. There are 28 hospitals providing secondary care and 1136 primary health facilities (140 health centres, 78 clinics, 884 Community Health Planning and Services, 31 maternity homes, 3 polyclinics). The majority of the health facilities are public (89.4%) with some (8.8%) private. Since 2008, Ghana introduced a free maternal health policy that covers pregnant women giving birth in all public and some private health facilities [28].

Routine birth data

Eligible subjects were women with a facility-based birth record between 1st January and 31st December 2017. Women whose place of residence was missing or non-mappable were excluded from analysis. All public health facilities report aggregate counts of women using birthing services in the DHIMS (District Health Information Management Systems) but only secondary facilities capture individual women's birth records electronically in the Ghana Health Service's (GHS) managed DHIMS. For primary health facilities, birth data were extracted from manually written book registers using a data extraction form. In secondary facilities, birth data were downloaded from DHIMS. Patient flows were calculated between origins (women's place of residence) and destinations (health facility locations) in the routine birth data. Details of the health facility used and the place of residence obtained from the routine birth data were used to spatially link the demand population, travel times and quality of care metrics.

Demand population

The number of women aged 15 to 45 years was estimated from WorldPop's 100m resolution gridded age-sex disaggregated population projections for 2020 [29]. WorldPop develops the population estimates by disaggregating administrative unit-linked census data into building footprints using machine learning methods and a library of geospatial covariates. Satellite-derived building footprints ensure populations are assigned to grid cells where people are known to live. We identified the population of women for each residential place by least cost travel time, then summed population counts for each place of residence to estimate demand within residential place catchments. GHS provided the list of place names with geographic coordinates.

For peer review only

Preliminary model-fitting shows population demand disaggregated by place of residence better explained utilisation patterns than population demand at sub-district level (Supplemental appendix 1). Demand was therefore modelled by place of residence.

Maternal healthcare quality metrics

An SPA was conducted in August and September 2021 to collect data on health facility attributes. Health facilities averaging five births per month in 2017 were surveyed and their geographic coordinates collected -for spatial linkage- via mobile data collection software [30].

Ten care quality domains were created from SPA data and combined to construct a quality of maternal health care composite index. The domains are human resource capacity, EmONC signal functions, medicines, non-medical supplies, amenities and infrastructure, referral systems, staff morale, privacy, training, and Water, Sanitation and Hygiene (WASH).

Domains were normalised (0 to 1) using range standardisation to ensure none of the domains unduly influenced the summary composite index. Domain scores were unweighted as there is no published evidence of their relative effects on utilisation [31]. The ten domain scores were averaged to derive the final composite score [32]. The internal consistency of the domains was verified with Cronbach's alpha. The ten domains correlated well for analysis (Cronbach coefficient = 0.74 (95%CI 0.65-0.80)). The composite index was grouped into quintiles [33].

Additional indicators of maternal health quality were estimated and evaluated against the composite ten-dimension index. Two additional composite indices were derived from a principal component analysis. The first included human resource capacity, number of EmONC signal functions, medicines, non-medical supplies, amenities and infrastructure, and referral systems. The second component was based on staff morale, privacy, training, and WASH.

Travel time model

Topographical data were used to model journey times from place of residence to health facilities. Travel times were modelled as the least cost path over an impedance surface. An impedance surface is a gridded map layer depicting travel speed. Road networks and water bodies from OpenStreetMap (OSM), a global mapping platform [34], were

For peer review only

incorporated into this layer. We estimated travel by walking and multi-modal (walking and motorised journeys). The multi-modal model combined walking on traversable land cover classes (mapped by the European Space Agency at 10x10m, year 2020) [35] and motorised travel on roads. Walking speeds (

Supplemental appendix 2) were adapted from previous studies using similar models [36] [37].

Motorised speeds on roads were inferred from Global Positioning System tracks collected via mobile devices. During the SPA data collection, we tracked road networks (767km), and recorded their condition (good, bad, very bad) at the time of data collection. GHS drivers who frequently travel within the study area added similar tags (good, bad, very bad) to printed OSM maps. Subsequently, OSM volunteers digitised the paper maps, uploading these to the OSM platform. The average travel speeds were proportionally weighted by road class and condition (Supplemental appendix 3).

The land cover, roads and water bodies were combined into a gridded friction dataset value representing the feasibility of traversing the landscape. The Tobler function [38] and elevation data (Shuttle Radar Topographic Mission 30m) [39] were used to model terrain effects on walking speed.

Travel times were estimated to health facilities providing any birthing service and secondary CEmONC facilities. Travel times were categorised to show critical thresholds for obstetric emergencies [13 40], counting women living in each travel time zone.

Modelling birthing service utilisation

The gravity-type spatial interaction model (SIM) framework applied in this study follows the Wilson group of SIM models [41]. Spatial interaction models predict the movement of services, goods or persons between two locations [42]. Gravity models, based on Newton's gravitational law, use the mass of two objects and the distance between them to predict their spatial interaction flows. Unconstrained, origin-constrained and destination-constrained models were implemented in this study. The unconstrained model fits coefficient for facility quality, proximity and population demand. In contrast, the origin-constrained model replaces the population demand term with separate coefficients for each origin, whilst the destination-constrained model replaces facility attractiveness with separate coefficients for each destination.

For peer review only

Travel time, population demand and care quality predictors were included in the models due to their influence on birthing service utilisation. In LMICs, systematic review evidence shows that proximity to a health facility significantly increased facility-based births [43]. The population of women was included because more women of child-bearing age living near a health facility should increase births. Lastly, health service quality could affect birthing service utilisation at nearby health facilities [26]. We hypothesise that staff morale, privacy, training and wash quality dimensions would have a greater effect on birthing service utilisation than human resource capacity, number of EmONC signal functions provided, availability of medicines, non-medical supplies, amenities and infrastructure, and referral systems.

The predicted outcome is the number of women making trips from a residential community to a health facility. The data is a patient flow matrix between all residential places and health facilities. Therefore, there were zeros where no interactions occurred between a residential population and a health facility. Consequently, the Poisson model was over-dispersed (8.2, $z=6.35$, $p < 0.001$) and zero-inflated. Hence, a zero-inflated, negative binomial model was implemented to address overdispersion and excess zeros [44 45]. Travel time between place of origin and destination facility was used to predict excess zeros because women would usually not travel unreasonably long distances to give birth in a health facility. The independent variables were log transformed for linearity to facilitate SIM calibration in a regression framework [46]. To enhance interpretation, marginal predicted counts of utilisation were estimated from the unconstrained model. The incidence rate ratios of the constrained models were mapped. The Akaike Information Criterion (AIC) was used to evaluate the relative predictive importance of the quality care metrics and population demand estimates. The AIC was used to evaluate models [47]. The coefficient of determination and root mean square error were used to evaluate the final gravity models.

Ethics

This study received ethical approval from the University of Southampton (Ref: 54949.A1 and 54944) and the Ghana Health Service ethics review committee (Ref: GHS-ERC008/05/20). Informed consent was obtained from all participants in the service provision assessment. All methods were carried out in accordance with relevant guidelines and regulations.

For peer review only

Patient and public involvement

This research was done without patient involvement. Patients were not invited to comment on the study design and were not consulted to develop patient-relevant outcomes or interpret the results. Patients were not invited to contribute to the writing or editing of this document for readability or accuracy.

Results

There were 40911 women from 964 places of residence who gave birth in 131 health facilities included in the analysis. The majority (75.6%) gave birth in secondary facilities. Aggregate reports in DHIMS recorded births by 57018 women, of whom 47900 had corresponding individual records (DHIMS: 70%, paper register: 30%). Of these individual records, 42205 were geocoded (DHIMS: 73%, paper register: 27%).

Quality of maternal healthcare and demand for birthing services

Figure 1A shows the geographic distribution of women 15 to 45 years in the study area, representing demand for birthing services. There were 2000 or fewer women at most origins (84.1% of 964). Most districts had at least one highly populated place of residence, with an above average number of resident women. There were few (17(1.82%)) highly populated origins, mostly in Accra, with at least 10000 women.

Figure 1: A. Geographic distribution of women 15 to 45 years by place of residence (origins). B. Spatial distribution of health facilities by quality and level of care.

The average quality score for health facilities was 48%. The quality scores ranged from 12% to 82%. There was a 20% quality difference between the mean scores at primary (43.4%) and secondary health facilities (63.6%). Among primary health facilities, CHPS (39.6%) and maternity homes (37.8%) had the lowest average quality compared to health centres (44.7%) and polyclinics (45%). Approximately five health facilities per district provided birthing services, and the majority were health centres. Figure 1 B shows lower quality scores in primary health facilities and clustering of secondary health facilities in some urban areas.

For peer review only

Modelled travel time and population access to care

Travel times were estimated for all 853,085 resident women aged 15 to 45 years within Eastern Region. Over 50% of these women were within an hour's travel to a health facility offering any birthing service (Figure 2C). In contrast, most women were more than two hours' travel from CEmONC facilities providing lifesaving services such as blood transfusion and caesarean sections (Figure 2D). Less than 35% of women could walk or travel by mechanised transport to the nearest secondary care health facility with CEmONC services within the recommended two-hour threshold. Figure 2A and B shows the spatial inequalities in geographic access to birthing services. Most settlements could reach any form of birthing service within two hours (Figure 2A). However, only a few settlements could reach a higher quality CEmONC health facility capable of handling complications within that same time (Figure 2B). There is thus inadequate provision of CEmONC services, with most areas beyond two hours' travel.

Figure 2: Geographic distribution of multimodal (walking plus mechanised) travel time to (A) Any birthing service and (B) health facilities offering CEmONC; Percent and number of women 15 to 45 years living within walking and mechanised travel time thresholds to (C) any birthing service and (D) CEmONC health facilities (n=853,085).

Evaluating the influences on birthing service utilisation

The summary quality index calculated from the ten domains explained birthing service utilisation better than the other quality care indicators. Routine services comprising staff motivation, privacy, training and WASH had the least effect on birthing service use (Supplemental appendix 4).

Increasing travel time to birthing services decreased the count of women attending to give birth (Table 1). Standardised coefficients show that the count of women using birthing services decreased by 57.6% per standard deviation increase in travel time. In contrast, population demand for services and quality of care increased health service utilisation. Number of women attending for childbirth increased by 36.5% when the standard deviation of population demand increased by a unit. The effect of quality care on utilisation was higher than travel time and population, since a unit increase in the standard deviation of the care quality index doubled (208.3%) the count of women giving birth.

For peer review only

The destination-constrained model had the highest correlation between predicted and actual counts of women (R^2:10.6%). Although the unconstrained model reported the lowest correlation (5.5%), it had the lowest root mean square error (23.3). The origin-constrained model had the lowest AIC (Table 1).

Table 1: Incidence rate ratio estimates from a zero-inflated negative binomial model predicting number of women using birthing services (n=40911)

**Model**	**Unconstrained IRR (95% CI)**	**Origin constrained IRR (95% CI)**	**Destination constrained IRR (95% CI)**
Intercept	91.1 (75.74-109.57)	9.98 (0.72-137.67)	1.79 (0.93-3.44)
Log travel time in hours	0.3 (0.28-0.32)	0.16 (0.15-0.18)	0.21 (0.2-0.23)
Log number of women 15 to 45 years	1.3 (1.24-1.36)	-	1.26 (1.2-1.32)
Log quality care	140.2 (109.39-179.68)	703.02 (541.06-913.46)	-
Intercept	0.18 (0.14-0.23)	0.14 (0.1-0.18)	0.08 (0.06-0.1)
Log travel time	6.88 (6.19-7.65)	5.91 (5.24-6.68)	7.82 (6.87-8.9)
Model evaluation			
AIC	29444	27446	27765
*RMSE	23.3	357	147
*R Squared (%)	5.5	8.1	10.6

*RMSE (Root Mean Square Error) and R squared were estimated with the observed versus fitted number of women giving birth; IRR is incidence rate ratio

The average marginal effects, calculated from the unconstrained model, are presented in Figure 3. The estimates show a profound decrease in service utilisation within the first two hours' travel to facility, where utilisation drops by approximately 18% then marginally afterwards. Service utilisation gradually increases with population demand for services and then plateaus. Greater quality of maternal health care increased utilisation exponentially. The marginal effect of quality doubles from 1.5 to 2.5 women for a 10% increase in the quality of maternal care from 40%. From 50% to 100% quality, the effect on utilisation quadruples.

Figure 3: Predicted marginal effects on birthing service utilisation for (A) travel time, (B) demand for services and (C) quality of maternal health services based on the unconstrained model

The destination-constrained model effects for health facilities are mapped in Figure 4B. The reference health facility is a health centre scoring 47% maternal health quality. Compared to the reference health centre, CHPS facilities reduced the rate ratio of

For peer review only

utilisation by 57%, while a 35% increase was observed at health centres. Hospitals increased the utilisation rate ratio by a factor of 16.1. Just over half the health facilities (51.5%) had a statistically significant influence on utilisation. Of 30 hospitals, 25 (83.3%) significantly influenced birthing service utilisation compared to 42% of the 100 primary health facilities.

Higher residential population increased utilisation rate ratios. However, effects for most places of residence (534(58.1%)) did not significantly influence birthing service utilisation. Locations with lower rate ratio estimates were mostly insignificant, in contrast to locations with higher rate ratios (Figure 4). Furthermore, standard errors for estimates were higher at the locations with lower estimates.

Figure 4: Estimated incidence rate ratio effects at (A) residential town and (B) health facilities on the number of women using birthing services. Standard error of estimates at (C) residential town and (D) health facilities.

Discussion

The quality of maternal health services in health facilities had a greater effect on the use of birthing services than geographic accessibility. Including additional quality care dimensions incrementally explained more of the variation in birthing service utilisation There was a significant inverse effect between travel time and birthing service utilisation while increased population demand and quality of maternal health services promoted utilisation. Furthermore, many women lived beyond the World Health Organisation recommended two hour travel time thresholds to CEmONC health facilities [13 40].

Building on a previous study [27], the current study presents new findings and considerable methodological improvements. Compared to hospitals only in the previous analysis, the current analysis includes primary and secondary health facilities, increasing heterogeneity in utilisation and service quality. The SPA is current and use of modelled travel times improves on the straight-line distance in the previous study. Conceptually, travel time is more realistic than straight-line distance as it takes into

For peer review only

account topographical features such as elevation, rivers and road network. Lastly, the previous study calibrated an unconstrained model only, whereas the current study also estimates effects for individual origins and destinations.

Quality care effect and implications for maternal health

The findings in this study are consistent with the limited existing literature assessing healthcare quality and proximity's impacts on birthing service utilisation. In five African countries, maternal care quality was lower in primary health facilities than in secondary care facilities and higher quality was associated with higher utilisation rates [48]. In these five countries, standard linear regression modelling was used to predict quality care from birth volume, country, skilled staff per bed capacity and health facility ownership (public/private).

There is a lack of gravity-type SIMs in low and middle-income settings to facilitate methodological and result comparisons. Two studies in Ghana did not find a significant association between maternal care quality and health service utilisation [26 27]. Nesbitt and colleagues modelled utilisation to the nearest health facility. However, there is a high bypassing rate of health facilities in Ghana [27]. Nesbitt, et al. [26] argued that poor maternal health service quality metrics and homogeneity in service quality among health facilities could result in a weak relationship between health facility quality and utilisation. The other study did not observe significant variations in quality because they assessed only hospitals.

This study shows that health facilities with higher quality were associated with higher utilisation rates. A systematic review identifies characteristics of health facilities that satisfy women when they seek maternal health care in LMICs [22]. They found that women express higher satisfaction with maternal health services when there is sufficient infrastructure such as electricity, water and adequate bed capacity. Furthermore, the number of staff and their availability, particularly when women seek emergency obstetric care, determines service satisfaction [49]. Another critical factor for women is privacy during consultation, labour, and after birth [50].

Higher level of health facilities (primary/secondary) and health facility type (CHPS, health centre, hospital, etc) were associated with significantly greater utilisation, as were health facility capacity, routine care and EmONC signal functions. However, a

For peer review only

418 comprehensive ten domain quality index explained utilisation better than these simpler
419 measures, suggesting improving staff morale, privacy, WASH and other health facility
420 quality characteristics may also incrementally increase service utilisation. The SPA data
421 collection tool was constructed with questions from different maternal health quality
422 care tools to derive a broader composite index [51]. Therefore, our quality care index
423 composition is essential because inclusion of additional quality components
424 incrementally explained more variation in birthing service utilisation (see Supplemental
425 appendix 4).

426 Higher quality care promotes maternal health service utilisation but does not always
427 lead to the desired health outcomes [52]. For example, higher volumes of health facility
428 births did not reduce maternal and perinatal deaths in Ghana, but stillbirths were lower
429 at facilities with improved quality [4]. Hence, as efforts continue to increase skilled
430 attendance at birth, health facilities should be prepared to manage complications to
431 prevent maternal deaths.

432 Proximity effect and implications for maternal health

433 Although our current study did not quantify bypassing of health facilities, the utilisation
434 patterns were similar to our previous study [27], suggesting a high level of bypassing. Poor
435 women travelling from rural areas are most likely to bypass substandard health
436 facilities for a hospital [33]. Furthermore, Figure 1B shows the uneven distribution of
437 secondary care facilities. Whilst there are rural districts without hospitals, hospitals are
438 clustered in urban areas, leading to unequal geographic access to CEmONC in the region.

439 The clustering of higher quality maternal care in urban areas implies that rural women
440 in labour will travel further and spend more to receive better care. Consequently,
441 complications can result in death due to the longer travel times. Almost all the
442 ambulances for transporting patients in emergencies are in these urban hospitals.
443 Finally, the spatial distribution adds to the indirect costs of women travelling from rural
444 to urban health facilities [53].

445 Similar to our findings, several studies found geographic proximity to a facility increases
446 birthing service utilisation [54]. Higher quality health facilities with CEmONC were more
447 than two hours' travel away for most women. A study in 2012 shows approximately
448 63% women within two hours of CEmONC facilities in the study area, 19.1% higher than

For peer review only

our estimate [24]. The change in CEmONC coverage could be due to expansion of geographic coverage of CEmONC services not matching population growth or a decline in the quality of secondary facilities.

The extent of the geographic coverage of any birthing service implies improved access for uncomplicated births, but there is a high risk for complicated ones. Hence, some women in obstetric emergencies living beyond the two-hour critical threshold might die en route to a quality health facility with blood transfusion or surgical services [13 55]. Strategically upgrading some existing health facilities, particularly in rural areas, to provide CEmONC would reduce these inequalities in geographic access to quality healthcare.

The methods are transferable and scalable to settings with similar maternal health system structures, and data via health information systems. The findings are transferable to most regions in Ghana due to the similar health system structure. A key feature influencing healthcare-seeking travel by Ghanaian women is the unrestricted choice of health facilities [27], variations in quality and the no cost policy [28]. In Ghana, the cost of giving birth in public facilities is free under a national health insurance scheme at a minimal registration cost [28]. Thus, women might choose the best health facility near them if they can afford travel and other costs. Countries with similar financial models are likely to observe comparable patterns and similar effects of proximity and the service quality on birthing service utilisation.

The analysis does not cover women giving birth in underutilised health facilities because birth data was extracted from health facilities averaging five births per month, consistent with previous EmONC SPAs [56]. Also, women whose places of residence could not be linked with a geographic coordinate were excluded. However, there was only a marginal difference in geocoding success rates by health facility ownership type, reducing selection bias, as shown in Supplemental appendix 5. Aggregated data versus individual and geocoded data were close to the line-of-agreement for most health facilities (Supplemental appendix 6), suggesting individual registers are complete. Furthermore, the individual birth register records analysed are substantially more complete than in the previous study and, unlike this earlier study, includes all health facility tiers [27]. Data quality can be improved by scaling up digitisation of individual birth data in DHIMS to all health facilities and improving the documentation of place

For peer review only

481 names via reference datasets for geocoding to ensure uniform spatial scales. Since some women listed towns as their place of residence whilst others listed neighbourhoods within towns, the variable spatial precision of place names in urban areas might have affected model estimates. The study was also unable to account for individual characteristics that influence utilisation such as wealth and education.

486 The accuracy of the travel time estimates is contingent on the accuracy of input data such as road networks, travel speeds, land cover, elevation, and water bodies. Besides, transient geographic barriers such as impassable roads due to broken bridges, flood inundation, road diversions, and other challenges were not incorporated into the travel time estimates. In addition, transportation cost and availability can limit access to health facilities. Finally, modelled travel times often overestimate geographic access to care, relative to self-reported travel times from patient surveys [57]. We did not account for edge effects [58], the tendency for women in Eastern Region to use birthing services in other regions or vice versa. Some women with longer travel times within Eastern Region could thus travel shorter distances to nearby regions.

496 Birthing service demand was estimated from gridded population datasets derived from the Ghana 2010 census and other datasets [29]. The population estimates are disaggregated into building extents where people live, but this can underestimate populations in urban areas [59]. The higher error associated with the utilisation rate ratios in urban areas could be due to the population estimates and the variable geocoding precision in these areas.

502 This study does not seek to infer causality from the cross-sectional design as the birth data, SPA, and population estimates represent one period only.

504 Conclusion and future research

505 Since health service quality is associated with greater birthing service utilisation, our study suggests that increasing service quality may drive up utilisation as well as improving some health outcomes. A ten domain index incorporating service components such as staff morale and patient privacy better explained utilisation than facility capacity or range of services alone. Our finding implies these quality improvements could also lead to more modest utilisation increases. To increase the use of birthing services, higher quality health facilities should be sited closer to women,

For peer review only

particularly in rural areas. Since this cross-sectional study relies on routine data which has improved in completeness and quality over time, in future, it should be possible to assess how changes in service quality or geographic coverage affect utilisation through spatio-temporal analysis of routine data. Further analysis can additional origin or destination characteristics such as health facility ownership or population ethnicity at origins.

Acknowledgement

We are grateful to the Ghana Health Service for making their data available for analysis. Furthermore, we extend our gratitude to the midwives, health directors and health information officers who supported this study in different capacities. Solomon Boamah, Patient Dodge, Doris Mantey, Julius Gafli, and James Otaniba Okoi assisted in the service provision assessment data collection. We express thanks to Ghana OpenStreetMap community, particularly Seth Enock Nyamador and Samuel Darkwah Manu for updating the free spatial data used in modelling the travel times and the Ghana Health Service drivers who annotated road conditions on paper maps. We acknowledge the Economic and Social Research Council for funding this study through the South Coast Doctoral Training Partnership.

Contributions

WDG and JW conceptualised and designed the study, WDG analysed the data and wrote the original draft manuscript; JW, AJT, ZM, VA and AO supervised the analysis and reporting. All authors revised and edited the manuscript. All authors read and approved the final manuscript.

Funding

The study was funded by the Economic and Social Research Council (ESRC), UK [grant number ES/P000673/1] through the South Coast Doctoral Training Partnership. The funder had no role in the design, data collection, analysis, interpretation of data and writing of the manuscript. The researchers were independent with no influence from the ESRC.

For peer review only

Competing interests

AO at the time of the study is the Deputy Director General of the Ghana Health Service. The Ghana Health Service generates and owns the birth data analysed in this study and is responsible for healthcare delivery in Ghana. WDG previously worked with the Ghana Health Service as a public health information officer until 2017. The other authors have no interests to declare.

There are no financial interests to declare.

Data availability

The demographic dataset analysed during the current study is openly available in the WoldPop repository, https://www.worldpop.org/

The service provision assessment dataset analysed during the current study are available from the correspondent author on reasonable request.

The birth datasets analysed during the current study are not publicly available due to confidentiality and data licencing restrictions from the Ghana Health Service. They can be obtained from the Ghana Health Service (https://www.ghs.gov.gh/contact-us) with reasonable request.

Spatial data on roads and rivers are openly available from OpenStreetMap available for download through Geofabrik (https://www.geofabrik.de/)

Landcover data is openly available from the European Space Agency (https://worldcover2020.esa.int/download)

References

1. Kruk ME, Lewis TP, Arsenault C, et al. Improving health and social systems for all children in LMICs: structural innovations to deliver high-quality services. *The Lancet* 2022;399(10337):1830-44. doi: 10.1016/S0140-6736(21)02532-0
2. Campbell OMR, Graham WJ. Strategies for reducing maternal mortality: getting on with what works. *The Lancet* 2006;368(9543):1284-99. doi: 10.1016/S0140-6736(06)69381-1
3. Bobo FT, Asante A, Woldie M, et al. Spatial patterns and inequalities in skilled birth attendance and caesarean delivery in sub-Saharan Africa. *BMJ Global Health* 2021;6(10):e007074. doi: 10.1136/bmjgh-2021-007074

For peer review only

4. Gabrysch S, Nesbitt RC, Schoeps A, et al. Does facility birth reduce maternal and perinatal mortality in Brong Ahafo, Ghana? A secondary analysis using data on 119 244 pregnancies from two cluster-randomised controlled trials. *The Lancet Global Health* 2019;7(8):e1074-e87. doi: 10.1016/S2214-109X(19)30165-2

5. Juran S, Broer PN, Klug SJ, et al. Geospatial mapping of access to timely essential surgery in sub-Saharan Africa. *BMJ Global Health* 2018;3(4):e000875. doi: 10.1136/bmjgh-2018-000875

6. Gabrysch S, Campbell OM. Still too far to walk: Literature review of the determinants of delivery service use. *BMC Pregnancy and Childbirth* 2009;9(1):34. doi: 10.1186/1471-2393-9-34

7. Thaddeus S, Maine D. Too far to walk: Maternal mortality in context. *Social Science & Medicine* 1994;38(8):1091-110. doi: http://dx.doi.org/10.1016/0277-9536(94)90226-7

8. Tanou M, Kishida T, Kamiya Y. The effects of geographical accessibility to health facilities on antenatal care and delivery services utilization in Benin: a cross-sectional study. *Reproductive Health* 2021;18(1):205. doi: 10.1186/s12978-021-01249-x

9. Hanson C, Cox J, Mbaruku G, et al. Maternal mortality and distance to facility-based obstetric care in rural southern Tanzania: a secondary analysis of cross-sectional census data in 226 000 households. *The Lancet Global Health* 2015;3(7):e387-e95. doi: https://doi.org/10.1016/S2214-109X(15)00048-0

10. Banke-Thomas A, Avoka CK-o, Gwacham-Anisiobi U, et al. Travel of pregnant women in emergency situations to hospital and maternal mortality in Lagos, Nigeria: a retrospective cohort study. *BMJ Global Health* 2022;7(4):e008604. doi: 10.1136/bmjgh-2022-008604

11. Treacy L, Bolkan HA, Sagbakken M. Distance, accessibility and costs. Decision-making during childbirth in rural Sierra Leone: A qualitative study. *PLOS ONE* 2018;13(2):e0188280. doi: 10.1371/journal.pone.0188280

12. Alaofe H, Lott B, Kimaru L, et al. Emergency Transportation Interventions for Reducing Adverse Pregnancy Outcomes in Low- and Middle-Income Countries: A Systematic Review. *Ann Glob Health* 2020;86(1):147-47. doi: 10.5334/aogh.2934

13. WHO, UNFPA, MSPH AMDD, et al. Monitoring emergency obstetric care: a handbook. Geneva: World Health Organization 2009.

14. Lundin R, Mariani I, Peven K, et al. Quality of routine health facility data used for newborn indicators in low- and middle-income countries: A systematic review. *Journal of Global Health* 2022;12:04019. doi: 10.7189/jogh.12.04019

15. Li M, Brodsky I, Geers E. Barriers to use of health data in low-and middleincome countries a review of the literature. North Carolina: United States Agency for International Development and Measure Evaluation, 2018.

16. Hounton S, Chapman G, Menten J, et al. Accessibility and utilisation of delivery care within a Skilled Care Initiative in rural Burkina Faso. *Tropical Medicine & International Health* 2008;13(s1):44-52. doi: https://doi.org/10.1111/j.1365-3156.2008.02086.x

17. Peet ED, Okeke EN. Utilization and quality: How the quality of care influences demand for obstetric care in Nigeria. *PLOS ONE* 2019;14(2):e0211500. doi: 10.1371/journal.pone.0211500

18. Hotchkiss DR, Krasovec K, Driss Zine-Eddine El-Idrissi M, et al. The role of user charges and structural attributes of quality on the use of maternal health

For peer review only

services in Morocco. *The International Journal of Health Planning and Management* 2005;20(2):113-35. doi: https://doi.org/10.1002/hpm.802
19. Chou VB, Walker N, Kanyangarara M. Estimating the global impact of poor quality of care on maternal and neonatal outcomes in 81 low- and middle-income countries: A modeling study. *PLOS Medicine* 2019;16(12):e1002990. doi: 10.1371/journal.pmed.1002990
20. Hung YW, Hoxha K, Irwin BR, et al. Using routine health information data for research in low- and middle-income countries: a systematic review. *BMC Health Services Research* 2020;20(1):790. doi: 10.1186/s12913-020-05660-1
21. Knight HE, Self A, Kennedy SH. Why Are Women Dying When They Reach Hospital on Time? A Systematic Review of the 'Third Delay'. *PLOS ONE* 2013;8(5):e63846. doi: 10.1371/journal.pone.0063846
22. Srivastava A, Avan BI, Rajbangshi P, et al. Determinants of women's satisfaction with maternal health care: a review of literature from developing countries. *BMC Pregnancy and Childbirth* 2015;15(1):97. doi: 10.1186/s12884-015-0525-0
23. Mubiri P, Kajjo D, Okuga M, et al. Bypassing or successful referral? A population-based study of reasons why women travel far for childbirth in Eastern Uganda. *BMC Pregnancy and Childbirth* 2020;20(1):497. doi: 10.1186/s12884-020-03194-2
24. Gething PW, Johnson FA, Frempong-Ainguah F, et al. Geographical access to care at birth in Ghana: a barrier to safe motherhood. *BMC Public Health* 2012;12(1):991. doi: 10.1186/1471-2458-12-991
25. Dotse-Gborgbortsi W, Tatem AJ, Alegana V, et al. Spatial inequalities in skilled attendance at birth in Ghana: a multilevel analysis integrating health facility databases with household survey data. *Tropical Medicine & International Health* 2020;25(9):1044-54. doi: https://doi.org/10.1111/tmi.13460
26. Nesbitt RC, Lohela TJ, Soremekun S, et al. The influence of distance and quality of care on place of delivery in rural Ghana. *Scientific Reports* 2016;6(1):30291. doi: 10.1038/srep30291
27. Dotse-Gborgbortsi W, Dwomoh D, Alegana V, et al. The influence of distance and quality on utilisation of birthing services at health facilities in Eastern Region, Ghana. *BMJ Global Health* 2020;4(Suppl 5):e002020. doi: 10.1136/bmjgh-2019-002020
28. Ministry of Health. The Ghana health sector 2008 programme of work. Accra: GoG, 2007.
29. Pezzulo C, Hornby GM, Sorichetta A, et al. Sub-national mapping of population pyramids and dependency ratios in Africa and Asia. *Scientific Data* 2017;4(1):170089. doi: 10.1038/sdata.2017.89
30. Kobo Inc. KoBotoolbox [Computer Sofware Application] 2022 [Available from: https://www.kobotoolbox.org/kobo/.
31. Greco S, Ishizaka A, Tasiou M, et al. On the Methodological Framework of Composite Indices: A Review of the Issues of Weighting, Aggregation, and Robustness. *Social Indicators Research* 2019;141(1):61-94. doi: 10.1007/s11205-017-1832-9
32. Countdown. Mind the gap: equity and trends in coverage of maternal, newborn, and child health services in 54 Countdown countries. *The Lancet* 2008;371(9620):1259-67. doi: 10.1016/S0140-6736(08)60560-7
33. Escamilla V, Calhoun L, Winston J, et al. The Role of Distance and Quality on Facility Selection for Maternal and Child Health Services in Urban Kenya. *Journal of Urban Health* 2018;95(1):1-12. doi: 10.1007/s11524-017-0212-8

For peer review only

34. OpenStreetMap Contributors. Open geospatial map data [available online] 2022 [Available from: https://www.openstreetmap.org/ accessed 12/03/2022.
35. Zanaga D, Van De Kerchove R, De Keersmaecker W, et al. ESA WorldCover 10 m 2020 v100. 2021
36. Ray N, Ebener S. AccessMod 3.0: computing geographic coverage and accessibility to health care services using anisotropic movement of patients. *International Journal of Health Geographics* 2008;7(1):63. doi: 10.1186/1476-072X-7-63
37. Alegana VA, Wright JA, Pentrina U, et al. Spatial modelling of healthcare utilisation for treatment of fever in Namibia. *International Journal of Health Geographics* 2012;11(1):6. doi: 10.1186/1476-072X-11-6
38. Tobler W. Three presentations on geographical analysis and modeling: National Center for Geographic Information and Analysis. *Santa Barbara, CA93106-4060: University of California, Santa Barbara* 1993
39. Farr TG, Rosen PA, Caro E, et al. The Shuttle Radar Topography Mission. *Reviews of Geophysics* 2007;45(2) doi: https://doi.org/10.1029/2005RG000183
40. WHO, Unicef. Monitoring emergency obstetric care: a handbook: Geneva: World Health Organization, 2009.
41. Wilson AG. A family of spatial interaction models, and associated developments. *Environment and Planning A* 1971;3(1):1-32.
42. Fotheringham AS. Spatial Interaction Models. In: Smelser NJ, Baltes PB, eds. International Encyclopedia of the Social & Behavioral Sciences. Oxford: Pergamon 2001:14794-800.
43. Tegegne TK, Chojenta C, Loxton D, et al. The impact of geographic access on institutional delivery care use in low and middle-income countries: Systematic review and meta-analysis. *PLOS ONE* 2018;13(8):e0203130. doi: 10.1371/journal.pone.0203130
44. Burger M, van Oort F, Linders G-J. On the Specification of the Gravity Model of Trade: Zeros, Excess Zeros and Zero-inflated Estimation. *Spatial Economic Analysis* 2009;4(2):167-90. doi: 10.1080/17421770902834327
45. Congdon P. Approaches to Modelling Overdispersion in the Analysis of Migration. *Environment and Planning A: Economy and Space* 1993;25(10):1481-510. doi: 10.1068/a251481
46. Fotheringham AS, O'Kelly ME. Spatial interaction models: formulations and applications. Dordrecht, The Netherlands: Kluwer Academic Publishers Dordrecht 1989.
47. Burnham KP, Anderson DR. Model selection and multimodel inference: A practical information-theoretic approach. 2nd ed: Springer New York 2002.
48. Kruk ME, Leslie HH, Verguet S, et al. Quality of basic maternal care functions in health facilities of five African countries: an analysis of national health system surveys. *The Lancet Global Health* 2016;4(11):e845-e55. doi: 10.1016/S2214-109X(16)30180-2
49. Sumankuuro J, Crockett J, Wang S. Perceived barriers to maternal and newborn health services delivery: a qualitative study of health workers and community members in low and middle-income settings. *BMJ Open* 2018;8(11):e021223. doi: 10.1136/bmjopen-2017-021223
50. Afaya A, Dzomeku VM, Baku EA, et al. Women's experiences of midwifery care immediately before and after caesarean section deliveries at a public Hospital in the Western Region of Ghana. *BMC Pregnancy and Childbirth* 2020;20(1):8. doi: 10.1186/s12884-019-2698-4

For peer review only

51. Gabrysch S, Civitelli G, Edmond KM, et al. New Signal Functions to Measure the Ability of Health Facilities to Provide Routine and Emergency Newborn Care. *PLOS Medicine* 2012;9(11):e1001340. doi: 10.1371/journal.pmed.1001340
52. Dettrick Z, Firth S, Jimenez Soto E. Do Strategies to Improve Quality of Maternal and Child Health Care in Lower and Middle Income Countries Lead to Improved Outcomes? A Review of the Evidence. *PLOS ONE* 2013;8(12):e83070. doi: 10.1371/journal.pone.0083070
53. Adatara P, Strumpher J, Ricks E. Exploring the reasons why women prefer to give birth at home in rural northern Ghana: a qualitative study. *BMC Pregnancy and Childbirth* 2020;20(1):500. doi: 10.1186/s12884-020-03198-y
54. Wong KLM, Benova L, Campbell OMR. A look back on how far to walk: Systematic review and meta-analysis of physical access to skilled care for childbirth in Sub-Saharan Africa. *PLOS ONE* 2017;12(9):e0184432. doi: 10.1371/journal.pone.0184432
55. Bailey P, Lobis S, Maine D, et al. Monitoring emergency obstetric care: a handbook: World Health Organization 2009.
56. GHS. National assessment for emergency obstetric and newborn care, Ghana, 2011.
57. van Duinen AJ, Adde HA, Fredin O, et al. Travel time and perinatal mortality after emergency caesarean sections: an evaluation of the 2-hour proximity indicator in Sierra Leone. *BMJ Global Health* 2020;5(12):e003943. doi: 10.1136/bmjgh-2020-003943
58. Stewart Fotheringham A, Rogerson PA. GIS and spatial analytical problems. *International Journal of Geographical Information Systems* 1993;7(1):3-19. doi: 10.1080/02693799308901936
59. Fries B, Guerra CA, García GA, et al. Measuring the accuracy of gridded human population density surfaces: A case study in Bioko Island, Equatorial Guinea. *PLOS ONE* 2021;16(9):e0248646. doi: 10.1371/journal.pone.0248646

Supplementary material

Supplemental appendix 1: Comparison between demand aggregated at sub-district versus travel time to place of residence cost allocation

Supplemental appendix 2: Travel speed assigned to land cover and road types

Supplemental appendix 3: Proportionate weighted estimation of mechanised travel speeds by rood smoothness and road class

Supplemental appendix 4:Relative performance of quality care indices in predicting the count of women giving birth between town-health facilities

Supplemental appendix 5: Breakdown of data availability and missing data by health facility ownership

Supplemental appendix 6: Correlation between (A) Aggregated versus individual data, (B) Individual data versus geocoded individual data, and (C) Aggregated versus individual geocoded data

For peer review only

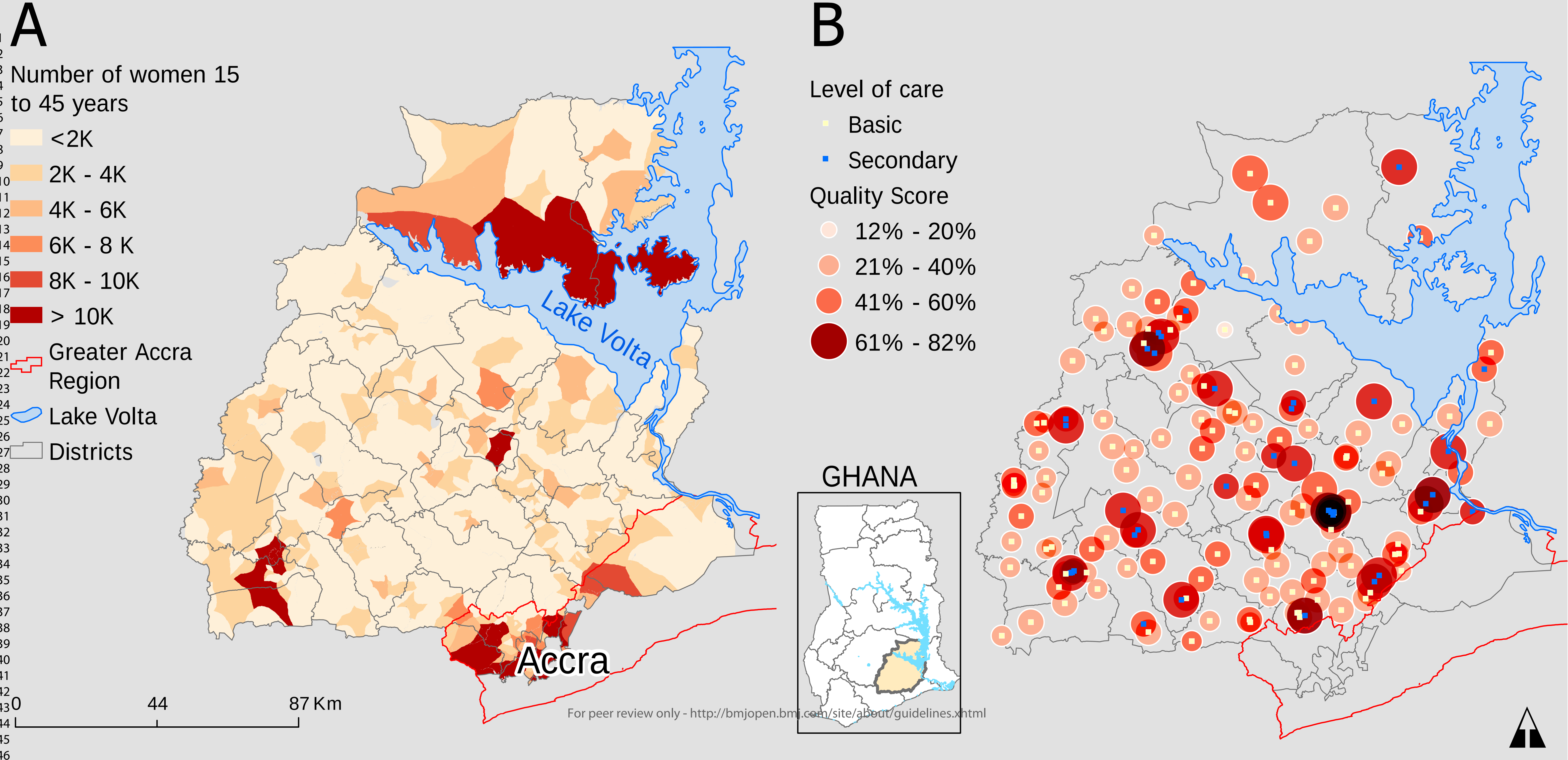

For peer review only - http://bmjopen.bmj.com/site/about/guidelines.xhtml

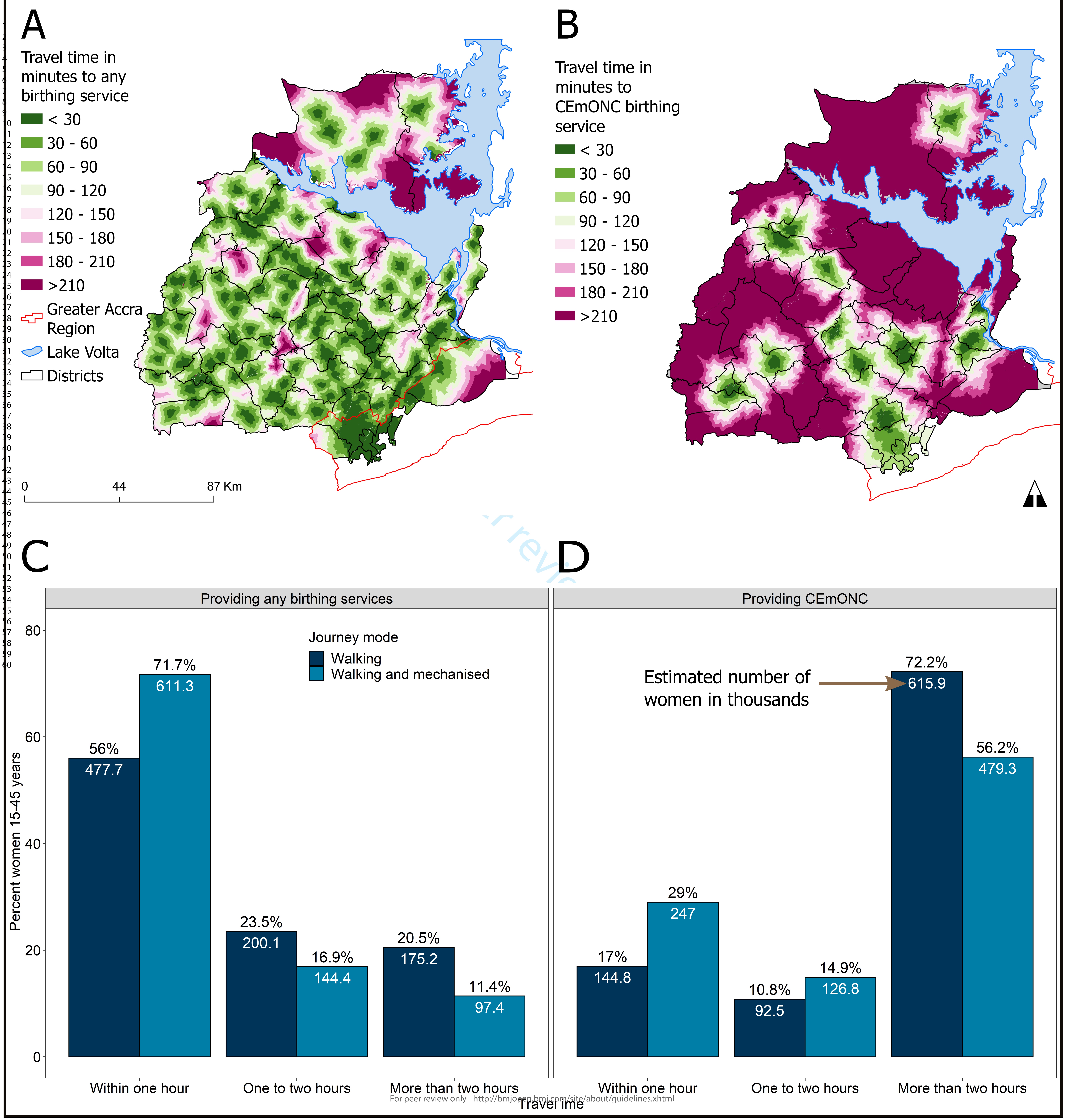
A
Travel time in minutes to any birthing service
< 30
30 - 60
60 - 90
90 - 120
120 - 150
150 - 180
180 - 210
>210
Greater Accra Region
Lake Volta
Districts
0
44
87 Km
B
Travel time in minutes to CEmONC birthing service
< 30
30 - 60
60 - 90
90 - 120
120 - 150
150 - 180
180 - 210
>210
C
D
Providing any birthing services
Providing CEmONC
Journey mode
Walking
Walking and mechanised
Estimated number of women in thousands
Percent women 15-45 years
0
20
40
60
80
56%
477.7
71.7%
611.3
23.5%
200.1
16.9%
144.4
20.5%
175.2
11.4%
97.4
17%
144.8
29%
247
10.8%
92.5
14.9%
126.8
72.2%
615.9
56.2%
479.3
Within one hour
One to two hours
More than two hours
Travel time

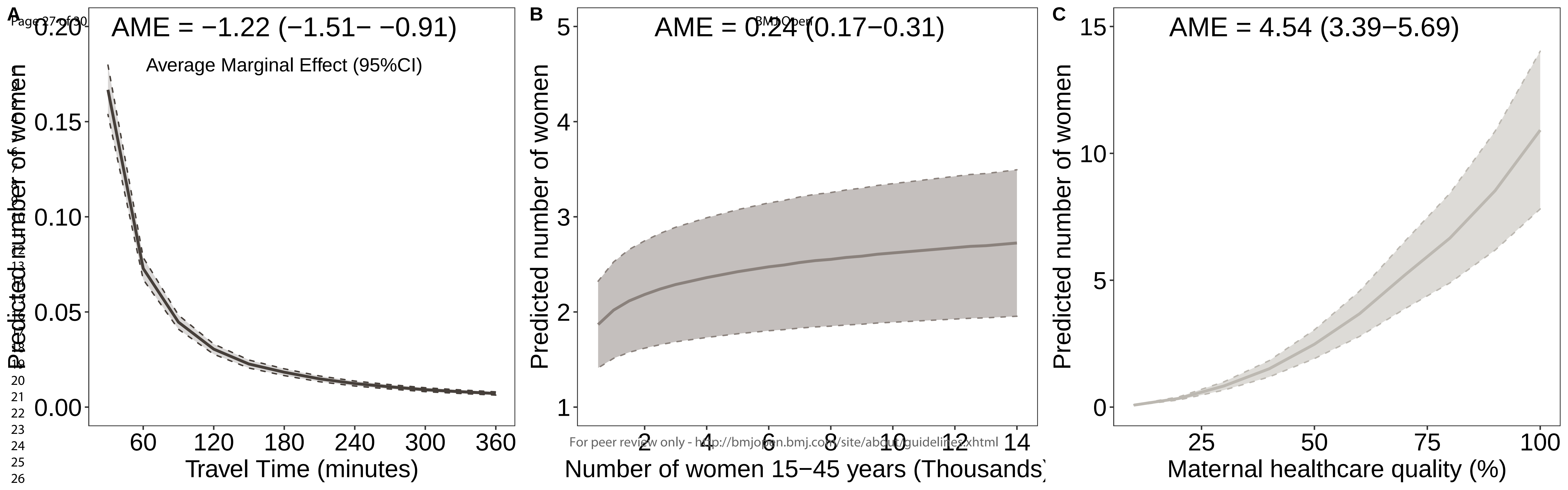
A
AME = −1.22 (−1.51− −0.91)
Average Marginal Effect (95%CI)
Predicted number of women
0.20
0.15
0.10
0.05
0.00
60
120
180
240
300
360
Travel Time (minutes)
B
AME = 0.24 (0.17−0.31)
Predicted number of women
5
4
3
2
1
2
4
6
8
10
12
14
Number of women 15−45 years (Thousands)
C
AME = 4.54 (3.39−5.69)
Predicted number of women
15
10
5
0
25
50
75
100
Maternal healthcare quality (%)

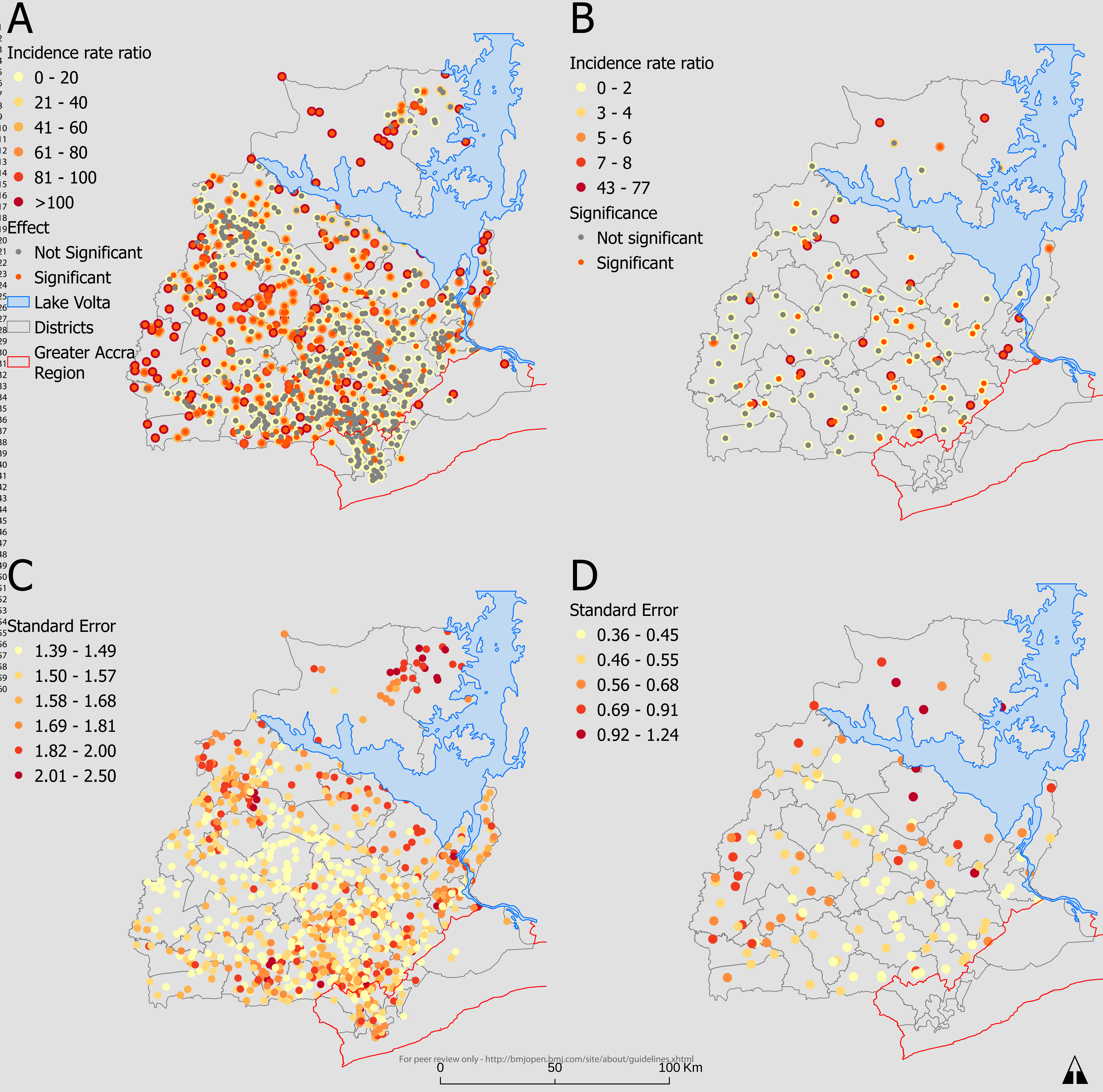
A
Incidence rate ratio
0 - 20
21 - 40
41 - 60
61 - 80
81 - 100
>100
Effect
Not Significant
Significant
Lake Volta
Districts
Greater Accra Region
B
Incidence rate ratio
0 - 2
3 - 4
5 - 6
7 - 8
43 - 77
Significance
Not significant
Significant
C
Standard Error
1.39 - 1.49
1.50 - 1.57
1.58 - 1.68
1.69 - 1.81
1.82 - 2.00
2.01 - 2.50
D
Standard Error
0.36 - 0.45
0.46 - 0.55
0.56 - 0.68
0.69 - 0.91
0.92 - 1.24
0
50
100 Km

Supplementary material

Supplemental appendix 1: Comparison between demand aggregated at sub-district versus travel time to place of residence cost allocation

Model	AIC	AIC Change
Travel time + summary quality index + number of women 15 to 45 years near the catchment of a town (origin) estimated by travel time to place of residence cost allocation.	84,796.6	0
Travel time + summary quality index + number of women 15 to 45 years in origin sub-district	92,298.1	7501.47

Supplemental appendix 2: Travel speed assigned to land cover and road types

Feature	Travel speeds (Kmh^{-1})	
	Multi modal	Walking
Land cover		
Grassland and built-up areas	5	5
Trees, shrubland, cropland, bare or sparse vegetation	4	4
Herbaceous wetland	3	3
Rivers and lakes	0	0
Roads		
Trunk	48	5
Primary	43	5
Secondary	40	5
Tertiary	35	5
Tracks and residential	30	5
Footpaths	5	5

Supplemental appendix 3: Proportionate weighted estimation of mechanised travel speeds by rood smoothness and road class

Road smoothness description by OSM	Average speed by road smoothness (A)	% primary (B)	A*B	% secondary (C)	A*C	% tertiary (D)	A*D	% track (E)	A*E	% trunk (F)	A*F	% All roads (G)	A*G
Horrible	20	0	0.0	2	0.3	1	0.2	9	1.8	0	0.0	4	0.7
Very bad	31	14	4.2	16	4.8	58	18.0	80	24.9	1	0.3	45	14.0
bad	31	16	5.1	30	9.2	19	5.9	9	2.9	1	0.3	14	4.2
good	48	65	31.0	49	23.6	16	7.6	1	0.4	98	46.9	35	16.7
intermediate	48	5	2.6	4	1.9	5	2.4	0	0.1	0	0.0	2	1.2
excellent	48	0	0.0	0	0.0	1	0.4	0	0.1	0	0.0	0	0.1
Speed applied to road types		100	43	100	40	100	35	100	30	100	48	100	37

For peer review only

For peer review only - http://bmjopen.bmj.com/site/about/guidelines.xhtml

The OSM road smoothness are described here: https://wiki.openstreetmap.org/wiki/Key:smoothness

Supplemental appendix 4:Relative performance of quality care indices in predicting the count of women giving birth between town-health facilities

**Model**	**AIC**	**AIC Change**
Travel time + number of women 15 to 45 years in origin sub-district + summary quality index (human resource capacity, signal functions, medicines, logistics, amenities, referral capacity, motivation, privacy, training and wash)	84,796.65	0
Travel time + number of women 15 to 45 years in origin sub-district + summary quality index quintiles	84,885.28	88.54
Travel time + number of women 15 to 45 years in origin sub-district + size of health facility (human resource capacity, signal functions, medicines, logistics, amenities, referral capacity)	84,328.42	502.76
Travel time + number of women 15 to 45 years in origin sub-district +health facility type (CHPS, health center, hospital, polyclinic, maternity home)	85,407.18	610.53
Travel time + number of women 15 to 45 years in origin sub-district + Number of EmONC signal functions	85,953.44	1156.79
Travel time + number of women 15 to 45 years in origin sub-district +health facility level (primary or secondary)	86,078.33	1281.68
Travel time + number of women 15 to 45 years in origin sub-district + routine quality (staff motivation, privacy, training, wash)	89,608.96	5478.0.03

For peer review only

Supplemental appendix 5: Breakdown of data availability and missing data by health facility ownership

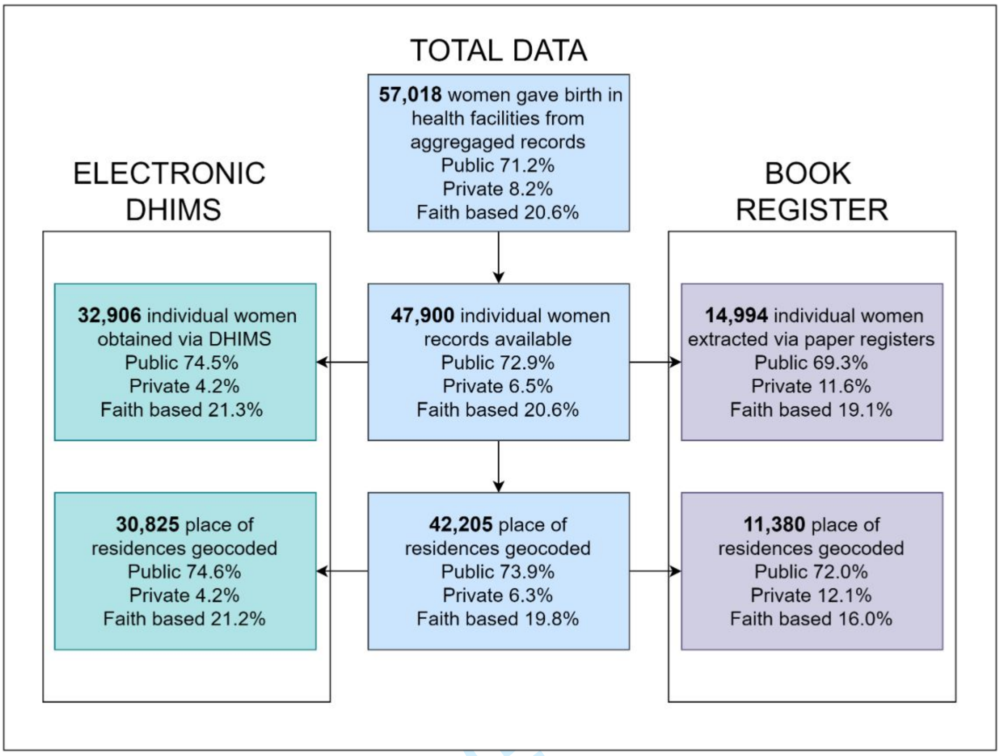

Supplemental appendix 6: Correlation between (A) Aggregated versus individual data, (B) Individual data versus geocoded individual data, and (C) Aggregated versus individual geocoded data

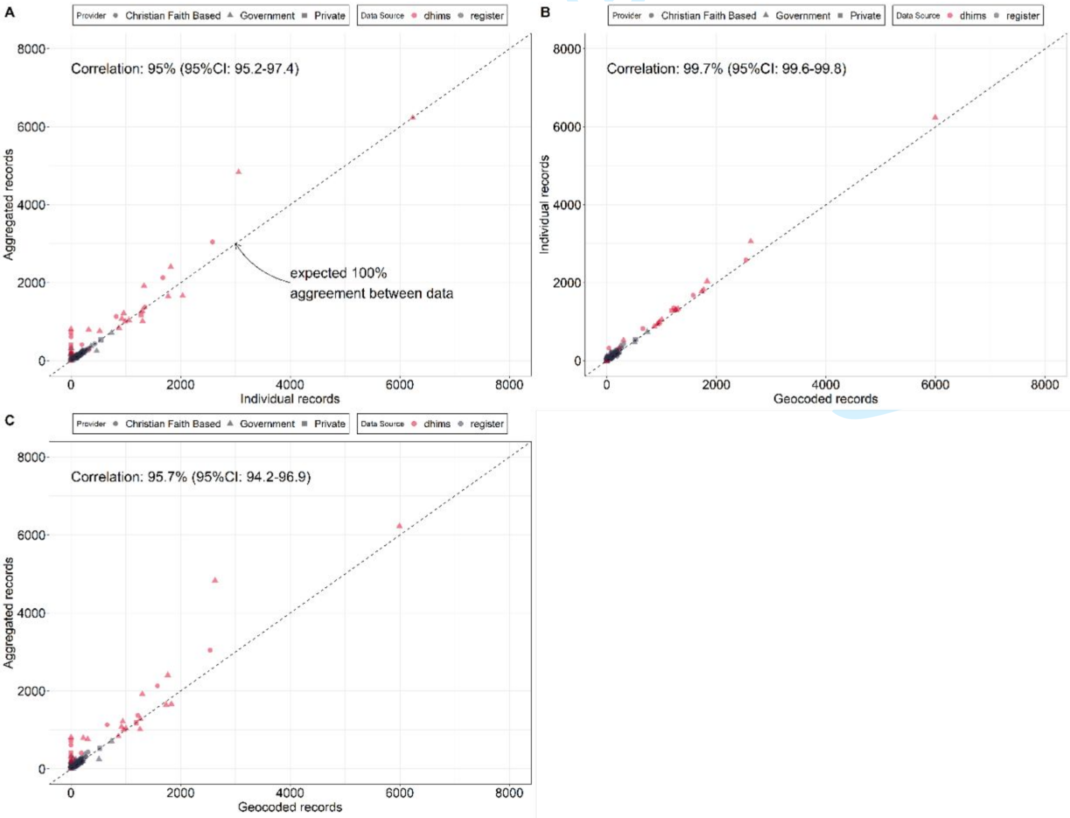

BMJ Open

Quality of maternal health care and travel time influence birthing service utilisation in Ghanaian health facilities: A geographic analysis of routine health data

Journal:	*BMJ Open*
Manuscript ID	bmjopen-2022-066792.R1
Article Type:	Original research
Date Submitted by the Author:	16-Nov-2022
Complete List of Authors:	Dotse-Gborgbortsi, Winfred; University of Southampton, School of Geography and Environmental Science; WorldPop, School of Geography and Environmental Science, University of Southampton<br>Tatem, Andrew; University of Southampton, School of Geography and Environmental Science; WorldPop, School of Geography and Environmental Science, University of Southampton<br>Matthews, Zoe; University of Southampton, Department of Social Statistics and Demography<br>Alegana, V; Kenya Medical Research Institute, Population Health Unit - Wellcome Trust Research Programme<br>Ofosu, Anthony; Ghana Health Service, Headquarters<br>Wright, Jim; University of Southampton, School of Geography and Environmental Science
**Primary Subject Heading**:	Health services research
Secondary Subject Heading:	Epidemiology, Global health, Health services research, Obstetrics and gynaecology, Public health
Keywords:	EPIDEMIOLOGY, Maternal medicine < OBSTETRICS, Health policy < HEALTH SERVICES ADMINISTRATION & MANAGEMENT, PUBLIC HEALTH

SCHOLARONE™
Manuscripts

I, the Submitting Author has the right to grant and does grant on behalf of all authors of the Work (as defined in the below author licence), an exclusive licence and/or a non-exclusive licence for contributions from authors who are: i) UK Crown employees; ii) where BMJ has agreed a CC-BY licence shall apply, and/or iii) in accordance with the terms applicable for US Federal Government officers or employees acting as part of their official duties; on a worldwide, perpetual, irrevocable, royalty-free basis to BMJ Publishing Group Ltd ("BMJ") its licensees and where the relevant Journal is co-owned by BMJ to the co-owners of the Journal, to publish the Work in this journal and any other BMJ products and to exploit all rights, as set out in our licence.

The Submitting Author accepts and understands that any supply made under these terms is made by BMJ to the Submitting Author unless you are acting as an employee on behalf of your employer or a postgraduate student of an affiliated institution which is paying any applicable article publishing charge ("APC") for Open Access articles. Where the Submitting Author wishes to make the Work available on an Open Access basis (and intends to pay the relevant APC), the terms of reuse of such Open Access shall be governed by a Creative Commons licence – details of these licences and which Creative Commons licence will apply to this Work are set out in our licence referred to above.

Other than as permitted in any relevant BMJ Author's Self Archiving Policies, I confirm this Work has not been accepted for publication elsewhere, is not being considered for publication elsewhere and does not duplicate material already published. I confirm all authors consent to publication of this Work and authorise the granting of this licence.

For peer review only

Quality of maternal health care and travel time influence birthing service utilisation in Ghanaian health facilities: A geographic analysis of routine health data

Winfred Dotse-Gborgbortsi [1,2], Andrew J Tatem [1,2], Zoë Matthews [3], Victor Alegana [4], Anthony Ofosu [5], Jim Wright [1].

[1] School of Geography and Environmental Science, University of Southampton, Southampton, UK

[2] WorldPop, School of Geography and Environmental Science, University of Southampton, Southampton, UK

[3] Department of Social Statistics and Demography, University of Southampton, Southampton, UK

[4] Population Health Unit, Kenya Medical Research Institute – Wellcome Trust Research Programme, Nairobi, Kenya

[5] Ghana Health Service, Headquarters, Accra, Ghana

Corresponding author: Winfred Dotse-Gborgbortsi, w.w.dotse-gborgbortsi@soton.ac.uk

For peer review only

Abstract

Introduction Quality of maternal health services and travels to health facilities both affect maternal health service utilisation, but few studies in Sub-Saharan Africa have assessed how service quality and proximity interact to affect utilisation. This study used geographic accessibility analysis to investigate how quality maternal health services and travel times interact to affect birthing service utilisation in Eastern Region, Ghana.

Methods This cross-sectional study uses a spatial interaction model to predict utilisation rates via a zero-inflated negative binomial spatial interaction gravity model. Through spatial linkage, the model integrated routinely collected birth records from health facilities, health facility service provision assessments, census-derived population estimates and travel time. Birth records for the period January to December 2017 were either manually extracted from handwritten registers or obtained from the Ghana Health Service District Information Management System..

Results As travel time from women's place of residence to the health facility increased up to two hours, the utilisation rate markedly decreased. Higher quality of maternal health services have a larger, positive effect on utilisation rates than service proximity. The quality of maternal health services was higher in hospitals than in primary care facilities. Most women (88.6%) travelling via mechanised transport were within two hours of any birthing service. The majority (56.2%) of women were beyond the two-hour threshold of critical comprehensive emergency obstetric and newborn care (CEmONC) services. Few CEmONC services were in urban centres, disadvantaging rural populations.

Conclusion To increase birthing service utilisation in Ghana, higher quality health facilities should be sited closer to women, particularly in rural areas. Beyond Ghana, routinely collected birth records could be used to understand the interaction of service proximity and quality.

Keywords: Geographic accessibility, maternal health, Health Information Systems, Geographic Information Systems, Health Services Accessibility,

For peer review only

Strengths and limitations of this study

- We integrated disparate data sources to estimate access, quality, and use of birthing services.
- We extracted patients' place of residence and destination facilities from routine health records, unlike most studies which use residential locations only from one-off household surveys
- Analysis excluded women whose place of residence could not be located, which could lead to selection bias.
- Due to data limitations, we could not account for individual socio-demographic characteristics that affect birthing service utilisation.

For peer review only

Introduction

Substandard maternal health quality in some health facilities in low and middle income countries (LMICs) has led to excess obstetric complications and maternal deaths [1]. Though quality maternal health care during labour is vital in reducing maternal mortality [2], and despite the steady rise in skilled birth coverage, maternity services remain below acceptable levels in most of sub-Saharan Africa [3]. The rising rate of health facility births is not associated with a decline in maternal deaths due to substandard health facilities incapable of averting maternal deaths [4]. Indeed, an estimated 92.5% of Sub-Saharan Africa's population is within two hours' journey time of a hospital [5] but maternal mortality is indicative of poor care.

There is ample evidence on the determinants of health facility utilisation for birth and the causes of maternal mortality [6 7]. Increasing distance to the closest health facility decreases the odds of health facility births [8] and increases maternal mortality rates [9]. For referred cases between health facilities too, the maternal health risk is higher for longer travel times [10]. Geographic accessibility hinders decisions to attend a health facility during pregnancy or seek care in a health facility for complications during home birth [11]. Furthermore, after deciding to use a health facility, proximity to care influences the choice of facility, journey time and transportation mode [12]. Therefore, to prevent maternal deaths, it is recommended that quality health facilities be within two hours' journey time of residential locations [13].

There is a paucity of studies using routine health data to evaluate the relationship between quality maternal health care and birthing service utilisation [14]. This is because the data can be incomplete, difficult to access, and expensive to extract and process [15 16]. Most studies conduct one-off sub-national surveys to collect data [17 18] or rely on national household surveys such as the Demographic and Health Survey [19 20]. However, surveys can be expensive and infrequent. Despite the challenges with routine health data, it has become increasingly important for malaria and maternal health research in Sub-Saharan Africa in recent years [21].

When women overcome geographic barriers to use birthing services, the quality of maternal health care on offer could affect the timeliness and adequacy of care. A review of supply-side barriers limiting access to quality maternal health care found drugs and equipment, staff capacity and morale, infrastructure and referral systems as some of the

For peer review only

challenges [22]. In addition, privacy during consultation and labour, cleanliness, and well-kept physical surroundings were other factors that determined service satisfaction among women seeking birthing services [23]. Although the Community Health Planning and Services (CHPS) initiative aims to achieve universal health coverage in Ghana, there is substandard maternal care in some CHPS facilities [24 25]. Thus, women are likely to travel longer to receive care in a health facility perceived as better than the nearest one [26].

There is inadequate evidence on healthcare utilisation and proximity to quality maternal care to support Ghana's maternal health interventions and programming. The latest analysis calculating journey times to different qualities of maternal health services is over a decade old [27]. Furthermore, the study measures potential access and did not estimate the effect of quality on utilisation rates. Two recent studies integrated quality care indicators estimated via service provision assessment (SPA) with secondary data analysis to predict the probability of skilled attendance at birth [28] and health facility births [29]. However, both studies estimated journeys to the nearest health facility, assuming women do not bypass health facilities to seek care elsewhere [30]. In the absence of detailed spatial information on where women reside and the actual health facilities they use, realised access modelling is challenging.

Building on previous work [30], this study aims to determine the effect of quality maternal health services and travel time on birthing service utilisation via geographic accessibility analysis by integrating routine birth data, SPA, and ancillary spatial data. Utilisation is modelled as a product of travel time from place of residence to health facilities, population demand in place of residence and maternal service quality in health facilities. Furthermore, the study aims to assess how different facility characteristics influence utilisation. Finally, it aims to estimate the number of women within 2 hours' walk or mechanised journey from any birthing service and from comprehensive emergency obstetric and newborn care services (CEmONC).

Methods

Research design and setting

The study is a cross-sectional spatial interaction analysis of birth service utilisation. Spatial interaction models predict the movement of people, goods or services from one

For peer review only

point to another [31]. The gravity models in this study predict the number of women making trips from a place of residence to a health facility to give birth as a function of travel times, the population of women, and the quality of maternal health services.

The study was conducted in Eastern Region, Ghana. The Eastern Region has 33 administrative districts divided into 225 sub-districts. The 2021 population census estimates 2.9 million persons in the region of which approximately 50.8% are females and 51.5% live in rural areas. There are 28 hospitals providing secondary care and 1136 primary health facilities (140 health centres, 78 clinics, 884 Community Health Planning and Services, 31 maternity homes, 3 polyclinics). The majority of the health facilities are public (89.4%) with some (8.8%) private. Since 2008, Ghana introduced a free maternal health policy that covers pregnant women giving birth in all public and some private health facilities [32].

Routine birth data

Eligible subjects were women with a facility-based birth record between 1st January and 31st December 2017. Women whose place of residence was missing or non-mappable were excluded from analysis. All public health facilities report aggregate counts of women using birthing services in the DHIMS (District Health Information Management Systems) but only secondary facilities capture individual women's birth records electronically in the Ghana Health Service's (GHS) managed DHIMS. For primary health facilities, birth data were extracted from manually written book registers using a data extraction form. In secondary facilities, birth data were downloaded from DHIMS. Patient flows were calculated between origins (women's place of residence) and destinations (health facility locations) in the routine birth data. Details of the health facility used and the place of residence obtained from the routine birth data were used to spatially link the demand population, travel times and quality of care metrics. The women's characteristics collected but not analysed are age, parity, level of education, and occupation.

Demand population

The number of women aged 15 to 45 years was estimated from WorldPop's 100m resolution gridded age-sex disaggregated population projections for 2020 [33]. WorldPop develops the population estimates by disaggregating administrative unit-linked census data into building footprints using machine learning methods and a library of geospatial

For peer review only

covariates. Satellite-derived building footprints ensure populations are assigned to grid cells where people are known to live. We identified the population of women for each residential place by least cost travel time, then summed population counts for each place of residence to estimate demand within residential place catchments. GHS provided the list of place names with geographic coordinates.

Preliminary model-fitting shows population demand disaggregated by place of residence better explained utilisation patterns than population demand at sub-district level (Supplemental appendix 1). Demand was therefore modelled by place of residence.

Maternal healthcare quality metrics

An SPA was conducted in August and September 2021 to collect data on health facility attributes. Health facilities averaging five births per month in 2017 were surveyed and their geographic coordinates collected -for spatial linkage- via mobile data collection software [34]. Out of the 1136 health facilities, 150 were eligible. However, 19 of the 150 eligible facilities were excluded because they could not provide individual-level routine birth register records for analysis. Thus, 131 health facilities were analysed.

Ten care quality domains were created from SPA data and combined to construct a quality of maternal health care composite index. The domains are human resource capacity, EmONC signal functions, medicines, non-medical supplies, amenities and infrastructure, referral systems, staff morale, privacy, training, and Water, Sanitation and Hygiene (WASH).

Domains were normalised (0 to 1) using range standardisation to ensure none of the domains unduly influenced the summary composite index. Domain scores were unweighted as there is no published evidence of their relative effects on utilisation [35]. The ten domain scores were averaged to derive the final composite score [36]. The internal consistency of the domains was verified with Cronbach's alpha. The ten domains correlated well for analysis (Cronbach coefficient = 0.74 (95%CI 0.65-0.80)). The composite index was grouped into quintiles [37].

Additional indicators of maternal health quality were estimated and evaluated against the composite ten-dimension index. Two additional composite indices were derived from a principal component analysis. The first included human resource capacity, number of EmONC signal functions, medicines, non-medical supplies, amenities and

For peer review only

infrastructure, and referral systems. The second component was based on staff morale, privacy, training, and WASH.

Travel time model

Topographical data were used to model journey times from place of residence to health facilities. Travel times were modelled as the least cost path over an impedance surface. An impedance surface is a gridded map layer depicting travel speed. Road networks and water bodies from OpenStreetMap (OSM), a global mapping platform [38], were incorporated into this layer. We estimated travel by walking and multi-modal (walking and motorised journeys). The multi-modal model combined walking on traversable land cover classes (mapped by the European Space Agency at 10x10m, year 2020) [39] and motorised travel on roads. Walking speeds (

Supplemental appendix 2) were adapted from previous studies using similar models [40] [41].

Motorised speeds on roads were inferred from Global Positioning System tracks collected via mobile devices. During the SPA data collection, we tracked road networks (767km), and recorded their condition (good, bad, very bad) at the time of data collection. GHS drivers who frequently travel within the study area added similar tags (good, bad, very bad) to printed OSM maps. Subsequently, OSM volunteers digitised the paper maps, uploading these to the OSM platform. The average travel speeds were proportionally weighted by road class and condition (Supplemental appendix 3).

The land cover, roads and water bodies were combined into a gridded friction dataset value representing the feasibility of traversing the landscape. The Tobler function [42] and elevation data (Shuttle Radar Topographic Mission 30m) [43 44] were used to model terrain effects on walking speed.

Travel times were estimated to health facilities providing any birthing service and secondary CEmONC facilities. Travel times were categorised to show critical thresholds for obstetric emergencies [13 45], counting women living in each travel time zone with the census-derived WorldPop gridded population of women 15 to 45 years.

Modelling birthing service utilisation

The gravity-type spatial interaction model (SIM) framework applied in this study follows the Wilson group of SIM models [46]. Spatial interaction models predict the

For peer review only

movement of services, goods or persons between two locations [31]. Gravity models, based on Newton's gravitational law, use the mass of two objects and the distance between them to predict their spatial interaction flows. Unconstrained, origin-constrained and destination-constrained models were implemented in this study. The unconstrained model fits coefficient for facility quality, proximity and population demand. In contrast, the origin-constrained model replaces the population demand term with separate coefficients for each origin, whilst the destination-constrained model replaces facility attractiveness with separate coefficients for each destination.

Travel time, population demand and care quality predictors were included in the models due to their influence on birthing service utilisation. In LMICs, systematic review evidence shows that proximity to a health facility significantly increased facility-based births [47]. The population of women was included because more women of child-bearing age living near a health facility should increase births. Lastly, health service quality could affect birthing service utilisation at nearby health facilities [29]. We hypothesise that staff morale, privacy, training and wash quality dimensions would have a greater effect on birthing service utilisation than human resource capacity, number of EmONC signal functions provided, availability of medicines, non-medical supplies, amenities and infrastructure, and referral systems.

The predicted outcome is the number of women making trips from a residential community to a health facility. The data is a patient flow matrix between all residential places and health facilities. Therefore, there were zeros where no interactions occurred between a residential population and a health facility. Consequently, the Poisson model was over-dispersed (8.2, z=6.35, $p < 0.001$) and zero-inflated. Hence, a zero-inflated, negative binomial model was implemented to address overdispersion and excess zeros [48 49]. Travel time between place of origin and destination facility was used to predict excess zeros because women would usually not travel unreasonably long distances to give birth in a health facility. The independent variables were log transformed for linearity to facilitate SIM calibration in a regression framework [50]. To enhance interpretation, we relied on marginal predicted counts to estimate utilisation and the interaction between quality and travel time from the unconstrained model. Likewise, we standardised the model estimates for easier interpretation. The incidence rate ratios of the constrained models were mapped. The Akaike Information Criterion (AIC) was

For peer review only

used to evaluate the relative predictive importance of the quality care metrics and population demand estimates. The AIC was used to evaluate models [51]. The coefficient of determination and root mean square error were used to evaluate the final gravity models.

Ethics

This study received ethical approval from the University of Southampton (Ref: 54949.A1 and 54944) and the Ghana Health Service ethics review committee (Ref: GHS-ERC008/05/20). Informed consent was obtained from all participants in the service provision assessment. All methods were carried out in accordance with relevant guidelines and regulations.

Patient and public involvement

This research was done without patient involvement. Patients were not invited to comment on the study design and were not consulted to develop patient-relevant outcomes or interpret the results. Patients were not invited to contribute to the writing or editing of this document for readability or accuracy.

Results

There were 40911 women from 964 places of residence who gave birth in 131 health facilities included in the analysis. The majority (75.6%) gave birth in secondary facilities. Aggregate reports in DHIMS recorded births by 57018 women, of whom 47900 had corresponding individual records (DHIMS: 70%, paper register: 30%). Of these individual records, 42205 were geocoded (DHIMS: 73%, paper register: 27%).

Quality of maternal healthcare and demand for birthing services

Figure 1A shows the geographic distribution of women 15 to 45 years in the study area, representing demand for birthing services. There were 2000 or fewer women at most origins (84.1% of 964). Most districts had at least one highly populated place of residence, with an above average number of resident women. There were few (17(1.82%)) highly populated origins, mostly in Accra, with at least 10000 women.

Figure 1: (A) Geographic distribution of women 15 to 45 years by place of residence (origins). (B) Spatial distribution of health facilities by quality and level of care.

For peer review only

The average quality score for health facilities was 48%. The quality scores ranged from 12% to 82%. There was a 20% quality difference between the mean scores at primary (43.4%) and secondary health facilities (63.6%). Among primary health facilities, CHPS (39.6%) and maternity homes (37.8%) had the lowest average quality compared to health centres (44.7%) and polyclinics (45%). Approximately five health facilities per district provided birthing services, and the majority were health centres. Figure 1 B shows lower quality scores in primary health facilities and clustering of secondary health facilities in some urban areas.

Modelled travel time and population access to care

Figure 2 shows the spatial inequalities in geographic access to birthing services. Most settlements could reach any form of birthing service within two hours (Figure 2A). However, only a few settlements could reach a higher quality CEmONC health facility capable of handling complications within that same time (Figure 2B). There is thus inadequate provision of CEmONC services, with most areas beyond two hours' travel. Travel times were estimated for all 853,085 resident women aged 15 to 45 years within Eastern Region. Over 50% of these women were within an hour's travel to a health facility offering any birthing service (Figure 2C). In contrast, most women were more than two hours' travel from CEmONC facilities providing lifesaving services such as blood transfusion and caesarean sections (Figure 2D). Less than 35% of women could walk or travel by mechanised transport to the nearest secondary care health facility with CEmONC services within the recommended two-hour threshold.

Figure 2: Geographic distribution of multimodal (walking plus mechanised) travel time to (A) Any birthing service and (B) health facilities offering CEmONC; Percent and number of women 15 to 45 years living within walking and mechanised travel time thresholds to (C) any birthing service and (D) CEmONC health facilities (n=853,085).

Evaluating the influences on birthing service utilisation

The summary quality index calculated from the ten domains explained birthing service utilisation better than the other quality care indicators. Routine services comprising staff motivation, privacy, training and WASH had the least effect on birthing service use (Supplemental appendix 4).

For peer review only

The coefficients for the unconstrained model in Table 1 were transformed into incidence rate ratio (IRR) and standardised for easier interpretability. Higher travel time to birthing services decreased the count of women attending to give birth. Model results in Table 1 show that the count of women using birthing services decreased by 57.6% (IRR 0.3, 95%CI: 0.28-0.32) per standard deviation increase in travel time. In contrast, population demand for services and quality of care increased health service utilisation. Number of women attending for childbirth increased by 36.5% (IRR 1.3, 95%CI: 1.23-1.36) when the standard deviation of population demand increased by a unit. The effect of quality care on utilisation was higher than travel time and population. There was a 208.3% (IRR 140.19, 95%CI: 109.39-179.68) increase in the count of women given birth per unit increase in the standard deviation of the care quality index.

The destination-constrained model had the highest correlation between predicted and actual counts of women (R^2:10.6%). Although the unconstrained model reported the lowest correlation (5.5%), it had the lowest root mean square error (23.3). The origin-constrained model had the lowest AIC (Table 1).

Table 1: Incidence rate ratio estimates from a zero-inflated negative binomial model predicting number of women using birthing services (n=40911)

**Model**	**Unconstrained IRR (95% CI)**	**Origin constrained IRR (95% CI)**	**Destination constrained IRR (95% CI)**
Intercept	91.1 (75.74-109.57)	9.98 (0.72-137.67)	1.79 (0.93-3.44)
Log travel time in hours	0.30 (0.28-0.32)	0.16 (0.15-0.18)	0.21 (0.2-0.23)
Log number of women 15 to 45 years	1.30 (1.24-1.36)	-	1.26 (1.2-1.32)
Log quality care	140.20 (109.39-179.68)	703.02 (541.06-913.46)	-
Intercept	0.18 (0.14-0.23)	0.14 (0.1-0.18)	0.08 (0.06-0.1)
Log travel time	6.88 (6.19-7.65)	5.91 (5.24-6.68)	7.82 (6.87-8.9)
Model evaluation			
AIC	29444	27446	27765
*RMSE	23.3	357	147
*R Squared (%)	5.5	8.1	10.6

*RMSE (Root Mean Square Error) and R squared were estimated with the observed versus fitted number of women giving birth; IRR is incidence rate ratio

The average marginal effects, calculated from the unconstrained model, are presented in Figure 3. The estimates show a profound decrease in service utilisation within the first two hours' travel to facility, where utilisation drops from 0.073 (95% CI: 0.067 –

0.079) at one hour to 0.030 (95% CI: 0.028 – 0.033) at two hours (Figure 3A). Thereafter, the change in the predicted number of women was marginal from three hours and beyond. Service utilisation gradually increases with population demand for services and then plateaus (Figure 3B). Greater quality of maternal health care increased utilisation exponentially (Figure 3C). For a 10% increase in quality from 40% to 50%, the marginal effect doubles from 1.51 (95% CI: 1.19 – 1.83) to 2.47 (95% CI: 1.91 – 3.04) women, respectively. From 50% to 100% quality, the effect on utilisation quadruples to 10.91 (95% CI: 7.80 – 14.03). Given the same quality, the number of predicted women are significantly higher at lower travel times as shown in the interaction marginal effects plot (Figure 3D). Furthermore, the interactions do not have a significant effect beyond the critical two-hour threshold.

Figure 3: Predicted marginal effects on birthing service utilisation for (A) travel time, (B) demand for services and (C) quality of maternal health services, (D) interacting quality and travel time effect based on the unconstrained model.

Higher residential population increased utilisation rate ratios. However, effects for most places of residence (534(58.1%)) did not significantly influence birthing service utilisation. Residential locations with lower rate ratio estimates were mostly insignificant, in contrast to residential locations with higher rate ratios (Figure 4A). Furthermore, standard errors for estimates were higher at the residential locations with lower estimates.

The destination-constrained model effects for health facilities are mapped in Figure 4B. The reference health facility is had the median quality score (47%) and is a health centre. Compared to the reference health centre, CHPS facilities with relatively lower quality scores reduced the rate ratio of utilisation by 0.57 (95% CI: 0.17 – 2.25), while a 1.35 (95% CI: 0.38 – 5.43) increase was observed at health centres. Hospitals increased the utilisation rate ratio by a factor of 16.15 (95%CI: 7.18 – 36.56). Just over half the health facilities (51.5%) had a statistically significant influence on utilisation. Of 30 hospitals, 25 (83.3%) significantly influenced birthing service utilisation compared to 42% of the 100 primary health facilities. There was relatively higher error at locations with lower incidence rates and vice versa (Figure 4C and Figure 4D).

Figure 4: Estimated incidence rate ratio effects at (A) residential town and (B) health facilities on the number of women using birthing services. Standard error of estimates at (C) residential town and (D) health facilities.

For peer review only

Discussion

The quality of maternal health services in health facilities had a greater effect on the use of birthing services than geographic accessibility. Including additional quality care dimensions incrementally explained more of the variation in birthing service utilisation There was a significant inverse effect between travel time and birthing service utilisation while increased population demand and quality of maternal health services promoted utilisation. Furthermore, many women lived beyond the World Health Organisation recommended two hour travel time thresholds to CEmONC health facilities [13 45].

Whilst most studies analysing the use of birthing services in health facilities have relied on cross-sectional surveys [29 52], only one recent study has used routine HIMIS data collected on an ongoing basis [30]. Relative to this earlier study, we develop the use of routine HMIS data by analysing patient flows from primary and secondary care levels with updated SPA and improved geographic accessibility estimates. Also, the earlier study calibrated an unconstrained gravity model only, whereas the current study also estimates effects for individual origins and destinations.

Quality care effect and implications for maternal health

The findings in this study are consistent with the limited existing literature assessing healthcare quality and proximity's impacts on birthing service utilisation. In five African countries, maternal care quality was lower in primary health facilities than in secondary care facilities and higher quality was associated with higher utilisation rates [53]. In these five countries, standard linear regression modelling was used to predict quality care from birth volume, country, skilled staff per bed capacity and health facility ownership (public/private).

There is a lack of gravity-type SIMs in low and middle-income settings to facilitate methodological and result comparisons. Two studies in Ghana did not find a significant association between maternal care quality and health service utilisation [29 30]. Nesbitt and colleagues modelled utilisation to the nearest health facility. However, there is a high bypassing rate of health facilities in Ghana [30]. Nesbitt, et al. [29] argued that poor maternal health service quality metrics and homogeneity in service quality among

For peer review only

health facilities could result in a weak relationship between health facility quality and utilisation. The other study did not observe significant variations in quality because they assessed only hospitals.

This study shows that health facilities with higher quality were associated with higher utilisation rates. A systematic review identifies characteristics of health facilities that satisfy women when they seek maternal health care in LMICs [23]. They found that women express higher satisfaction with maternal health services when there is sufficient infrastructure such as electricity, water and adequate bed capacity. Furthermore, the number of staff and their availability, particularly when women seek emergency obstetric care, determines service satisfaction [54]. Another critical factor for women is privacy during consultation, labour, and after birth [55].

Higher level of health facilities (primary/secondary) and health facility type (CHPS, health centre, hospital, etc) were associated with significantly greater utilisation, as were health facility capacity, routine care and EmONC signal functions. However, a comprehensive ten domain quality index explained utilisation better than these simpler measures, suggesting improving staff morale, privacy, WASH and other health facility quality characteristics may also incrementally increase service utilisation. The SPA data collection tool was constructed with questions from different maternal health quality care tools to derive a broader composite index [56]. Therefore, our quality care index composition is essential because inclusion of additional quality components incrementally explained more variation in birthing service utilisation (see Supplemental appendix 4).

Higher quality care promotes maternal health service utilisation but does not always lead to the desired health outcomes [57]. For example, higher volumes of health facility births did not reduce maternal and perinatal deaths in Ghana, but stillbirths were lower at facilities with improved quality [4]. Hence, as efforts continue to increase skilled attendance at birth, health facilities should be prepared to manage complications to prevent maternal deaths.

Proximity effect and implications for maternal health

Although our current study did not quantify bypassing of health facilities, the utilisation patterns were similar to our previous study [30], suggesting a high level of bypassing. Poor

For peer review only

women travelling from rural areas are most likely to bypass substandard health facilities for a hospital [37]. Furthermore, our map of health facilities shows the uneven distribution of secondary care facilities. Whilst there are rural districts without hospitals, hospitals are clustered in urban areas, leading to unequal geographic access to CEmONC in the region.

The clustering of higher quality maternal care in urban areas implies that rural women in labour will travel further and spend more to receive better care. Consequently, complications can result in death due to the longer travel times. Almost all the ambulances for transporting patients in emergencies are in these urban hospitals. Finally, the spatial distribution adds to the indirect costs of women travelling from rural to urban health facilities [58].

Similar to our findings, several studies found geographic proximity to a facility increases birthing service utilisation [59]. Higher quality health facilities with CEmONC were more than two hours' travel away for most women. A study in 2012 shows approximately 63% women within two hours of CEmONC facilities in the study area, 19.1% higher than our estimate [27]. The change in CEmONC coverage could be due to expansion of geographic coverage of CEmONC services not matching population growth or a decline in the quality of secondary facilities.

The extent of the geographic coverage of any birthing service implies improved access for uncomplicated births, but there is a high risk for complicated ones. Hence, some women in obstetric emergencies living beyond the two-hour critical threshold might die en route to a quality health facility with blood transfusion or surgical services [13 60]. The two hour recommend travel time [13] is relevant because bleeding is the leading direct cause of maternal mortality in sub-saharan Africa [61]. Strategically upgrading some existing health facilities, particularly in rural areas, to provide CEmONC would reduce these inequalities in geographic access to quality healthcare. While there are calls for expanding access to essential obstetric care, the demand should be carefully considered to ensure these specialised services such as surgery and blood transfusion are not underutilised [62].

The methods are transferable and scalable to settings with similar maternal health system structures, and data via health information systems. The findings are

For peer review only

transferable to most regions in Ghana due to the similar health system structure. A key feature influencing healthcare-seeking travel by Ghanaian women is the unrestricted choice of health facilities [30], variations in quality and the no cost policy [32]. In Ghana, the cost of giving birth in public facilities is free under a national health insurance scheme at a minimal registration cost [32]. Thus, women might choose the best health facility near them if they can afford travel and other costs. Countries with similar financial models are likely to observe comparable patterns and similar effects of proximity and the service quality on birthing service utilisation.

The analysis does not cover women giving birth in underutilised health facilities because birth data was extracted from health facilities averaging five births per month, consistent with previous EmONC SPAs [63]. Birth records were not available from 19 health facilities; the births in these facilities are 6.5% of total births, which poses some bias. Also, women whose places of residence could not be linked with a geographic coordinate were excluded. However, there was only a marginal difference in geocoding success rates by health facility ownership type, suggesting potential selection bias, as shown in Supplemental appendix 5. Aggregated data versus individual and geocoded data were close to the line-of-agreement for most health facilities (Supplemental appendix 6), suggesting individual registers are complete. Furthermore, the individual birth register records analysed are substantially more complete than in the previous study and, unlike this earlier study, includes all health facility tiers [30]. Data quality can be improved by scaling up digitisation of individual birth data in DHIMS to all health facilities and improving the documentation of place names via reference datasets for geocoding to ensure uniform spatial scales. Since some women listed towns as their place of residence whilst others listed neighbourhoods within towns, the variable spatial precision of place names in urban areas might have affected model estimates. The study was also unable to account for individual characteristics that influence utilisation such as wealth and education.

The accuracy of the travel time estimates is contingent on the accuracy of input data such as road networks, travel speeds, land cover, elevation, and water bodies. Besides, transient geographic barriers such as impassable roads due to broken bridges, flood inundation, road diversions, and other challenges were not incorporated into the travel time estimates. In addition, transportation cost and availability can limit access to

For peer review only

health facilities. Finally, modelled travel times often overestimate geographic access to care, relative to self-reported travel times from patient surveys [64]. We did not account for edge effects [65], the tendency for women in Eastern Region to use birthing services in other regions or vice versa. Some women with longer travel times within Eastern Region could thus travel shorter distances to nearby regions.

Birthing service demand was estimated from gridded population datasets derived from the Ghana 2010 census and other datasets [66]. The population estimates are disaggregated into building extents where people live, but this can underestimate populations in urban areas [67]. The higher error associated with the utilisation rate ratios in urban areas could be due to the population estimates and the variable geocoding precision in these areas.

This study does not seek to infer causality from the cross-sectional design as the birth data, SPA, and population estimates represent one period only.

Conclusion and future research

Since health service quality is associated with greater birthing service utilisation, our study suggests that increasing service quality may drive up utilisation as well as improving some health outcomes. A ten domain index incorporating service components such as staff morale and patient privacy better explained utilisation than facility capacity or range of services alone. Our finding implies these quality improvements could also lead to more modest utilisation increases. To increase the use of birthing services, higher quality health facilities should be sited closer to women, particularly in rural areas. Since this cross-sectional study relies on routine data which has improved in completeness and quality over time, in future, it should be possible to assess how changes in service quality or geographic coverage affect utilisation through spatio-temporal analysis of routine data. Having identified substantial bypassing in our analysis, in a future study, we plan to investigate facility by-passing as an outcome in relation to facility-level characteristics and the limited individual-level characteristics that are recorded on patient registers via a multilevel framework. Further analysis can include additional origin or destination characteristics such as health facility ownership or population ethnicity at origins.

For peer review only

Acknowledgement

We are grateful to the Ghana Health Service for making their data available for analysis. Furthermore, we extend our gratitude to the midwives, health directors and health information officers who supported this study in different capacities. Solomon Boamah, Patient Dodge, Doris Mantey, Julius Gafli, and James Otaniba Okoi assisted in the service provision assessment data collection. We express thanks to Ghana OpenStreetMap community, particularly Seth Enock Nyamador and Samuel Darkwah Manu for updating the free spatial data used in modelling the travel times and the Ghana Health Service drivers who annotated road conditions on paper maps. We acknowledge the Economic and Social Research Council for funding this study through the South Coast Doctoral Training Partnership.

Contributions

WDG and JW conceptualised and designed the study, WDG analysed the data and wrote the original draft manuscript; JW, AJT, ZM, VA and AO supervised the analysis and reporting. All authors revised and edited the manuscript. All authors read and approved the final manuscript.

Funding

The study was funded by the Economic and Social Research Council (ESRC), UK [grant number ES/P000673/1] through the South Coast Doctoral Training Partnership. The funder had no role in the design, data collection, analysis, interpretation of data and writing of the manuscript. The researchers were independent with no influence from the ESRC.

Competing interests

AO at the time of the study is the Deputy Director General of the Ghana Health Service. The Ghana Health Service generates and owns the birth data analysed in this study and is responsible for healthcare delivery in Ghana. WDG previously worked with the Ghana Health Service as a public health information officer until 2017. The other authors have no interests to declare.

There are no financial interests to declare.

For peer review only

Data availability

The demographic dataset analysed during the current study is openly available in the WoldPop repository, https://www.worldpop.org/

The service provision assessment dataset analysed during the current study are available from the correspondent author on reasonable request.

The birth datasets analysed during the current study are not publicly available due to confidentiality and data licencing restrictions from the Ghana Health Service. They can be obtained from the Ghana Health Service (https://www.ghs.gov.gh/contact-us) with reasonable request.

Spatial data on roads and rivers are openly available from OpenStreetMap available for download through Geofabrik (https://www.geofabrik.de/)

Landcover data is openly available from the European Space Agency (https://worldcover2020.esa.int/download)

References

1. Kruk ME, Lewis TP, Arsenault C, et al. Improving health and social systems for all children in LMICs: structural innovations to deliver high-quality services. *The Lancet* 2022;399(10337):1830-44. doi: 10.1016/S0140-6736(21)02532-0
2. Campbell OMR, Graham WJ. Strategies for reducing maternal mortality: getting on with what works. *The Lancet* 2006;368(9543):1284-99. doi: 10.1016/S0140-6736(06)69381-1
3. Bobo FT, Asante A, Woldie M, et al. Spatial patterns and inequalities in skilled birth attendance and caesarean delivery in sub-Saharan Africa. *BMJ Global Health* 2021;6(10):e007074. doi: 10.1136/bmjgh-2021-007074
4. Gabrysch S, Nesbitt RC, Schoeps A, et al. Does facility birth reduce maternal and perinatal mortality in Brong Ahafo, Ghana? A secondary analysis using data on 119 244 pregnancies from two cluster-randomised controlled trials. *The Lancet Global Health* 2019;7(8):e1074-e87. doi: 10.1016/S2214-109X(19)30165-2
5. Juran S, Broer PN, Klug SJ, et al. Geospatial mapping of access to timely essential surgery in sub-Saharan Africa. *BMJ Global Health* 2018;3(4):e000875. doi: 10.1136/bmjgh-2018-000875
6. Gabrysch S, Campbell OM. Still too far to walk: Literature review of the determinants of delivery service use. *BMC Pregnancy and Childbirth* 2009;9(1):34. doi: 10.1186/1471-2393-9-34
7. Thaddeus S, Maine D. Too far to walk: Maternal mortality in context. *Social Science & Medicine* 1994;38(8):1091-110. doi: http://dx.doi.org/10.1016/0277-9536(94)90226-7
8. Tanou M, Kishida T, Kamiya Y. The effects of geographical accessibility to health facilities on antenatal care and delivery services utilization in Benin: a cross-

For peer review only

sectional study. *Reproductive Health* 2021;18(1):205. doi: 10.1186/s12978-021-01249-x
9. Hanson C, Cox J, Mbaruku G, et al. Maternal mortality and distance to facility-based obstetric care in rural southern Tanzania: a secondary analysis of cross-sectional census data in 226 000 households. *The Lancet Global Health* 2015;3(7):e387-e95. doi: https://doi.org/10.1016/S2214-109X(15)00048-0
10. Banke-Thomas A, Avoka CK-o, Gwacham-Anisiobi U, et al. Travel of pregnant women in emergency situations to hospital and maternal mortality in Lagos, Nigeria: a retrospective cohort study. *BMJ Global Health* 2022;7(4):e008604. doi: 10.1136/bmjgh-2022-008604
11. Treacy L, Bolkan HA, Sagbakken M. Distance, accessibility and costs. Decision-making during childbirth in rural Sierra Leone: A qualitative study. *PLOS ONE* 2018;13(2):e0188280. doi: 10.1371/journal.pone.0188280
12. Alaofe H, Lott B, Kimaru L, et al. Emergency Transportation Interventions for Reducing Adverse Pregnancy Outcomes in Low- and Middle-Income Countries: A Systematic Review. *Ann Glob Health* 2020;86(1):147-47. doi: 10.5334/aogh.2934
13. WHO, UNFPA, MSPH AMDD, et al. Monitoring emergency obstetric care: a handbook. Geneva: World Health Organization 2009.
14. Ahmadian L, Salehi F, Bahaadinbeigy K. Application of geographic information systems in maternal health: a scoping review. *East Mediterr Health J* 2020;26(11):1403-14. doi: 10.26719/emhj.20.095 [published Online First: 2020/11/24]
15. Lundin R, Mariani I, Peven K, et al. Quality of routine health facility data used for newborn indicators in low- and middle-income countries: A systematic review. *J Glob Health* 2022;12:04019. doi: 10.7189/jogh.12.04019
16. Li M, Brodsky I, Geers E. Barriers to use of health data in low-and middleincome countries a review of the literature. North Carolina: United States Agency for International Development and Measure Evaluation, 2018.
17. Hounton S, Chapman G, Menten J, et al. Accessibility and utilisation of delivery care within a Skilled Care Initiative in rural Burkina Faso. *Tropical Medicine & International Health* 2008;13(s1):44-52. doi: https://doi.org/10.1111/j.1365-3156.2008.02086.x
18. Peet ED, Okeke EN. Utilization and quality: How the quality of care influences demand for obstetric care in Nigeria. *PLOS ONE* 2019;14(2):e0211500. doi: 10.1371/journal.pone.0211500
19. Hotchkiss DR, Krasovec K, Driss Zine-Eddine El-Idrissi M, et al. The role of user charges and structural attributes of quality on the use of maternal health services in Morocco. *The International Journal of Health Planning and Management* 2005;20(2):113-35. doi: https://doi.org/10.1002/hpm.802
20. Chou VB, Walker N, Kanyangarara M. Estimating the global impact of poor quality of care on maternal and neonatal outcomes in 81 low- and middle-income countries: A modeling study. *PLOS Medicine* 2019;16(12):e1002990. doi: 10.1371/journal.pmed.1002990
21. Hung YW, Hoxha K, Irwin BR, et al. Using routine health information data for research in low- and middle-income countries: a systematic review. *BMC Health Services Research* 2020;20(1):790. doi: 10.1186/s12913-020-05660-1
22. Knight HE, Self A, Kennedy SH. Why Are Women Dying When They Reach Hospital on Time? A Systematic Review of the 'Third Delay'. *PLOS ONE* 2013;8(5):e63846. doi: 10.1371/journal.pone.0063846

For peer review only

23. Srivastava A, Avan BI, Rajbangshi P, et al. Determinants of women's satisfaction with maternal health care: a review of literature from developing countries. *BMC Pregnancy and Childbirth* 2015;15(1):97. doi: 10.1186/s12884-015-0525-0
24. Bassoumah B, Adam AM, Adokiya MN. Challenges to the utilization of Community-based Health Planning and Services: the views of stakeholders in Yendi Municipality, Ghana. *BMC Health Services Research* 2021;21(1):1223. doi: 10.1186/s12913-021-07249-8
25. Addi B, Doe B, Oduro-Ofori E. Towards quality primary health care: the dilemma of Community-Based Health Planning and Services (CHPS) in health service provision in Ghana. *Journal of Health Organization and Management* 2022;36(4):482-502. doi: 10.1108/JHOM-06-2021-0219
26. Mubiri P, Kajjo D, Okuga M, et al. Bypassing or successful referral? A population-based study of reasons why women travel far for childbirth in Eastern Uganda. *BMC Pregnancy and Childbirth* 2020;20(1):497. doi: 10.1186/s12884-020-03194-2
27. Gething PW, Johnson FA, Frempong-Ainguah F, et al. Geographical access to care at birth in Ghana: a barrier to safe motherhood. *BMC Public Health* 2012;12(1):991. doi: 10.1186/1471-2458-12-991
28. Dotse-Gborgbortsi W, Tatem AJ, Alegana V, et al. Spatial inequalities in skilled attendance at birth in Ghana: a multilevel analysis integrating health facility databases with household survey data. *Tropical Medicine & International Health* 2020;25(9):1044-54. doi: https://doi.org/10.1111/tmi.13460
29. Nesbitt RC, Lohela TJ, Soremekun S, et al. The influence of distance and quality of care on place of delivery in rural Ghana. *Scientific Reports* 2016;6(1):30291. doi: 10.1038/srep30291
30. Dotse-Gborgbortsi W, Dwomoh D, Alegana V, et al. The influence of distance and quality on utilisation of birthing services at health facilities in Eastern Region, Ghana. *BMJ Global Health* 2020;4(Suppl 5):e002020. doi: 10.1136/bmjgh-2019-002020
31. Fotheringham AS. Spatial Interaction Models. In: Smelser NJ, Baltes PB, eds. International Encyclopedia of the Social & Behavioral Sciences. Oxford: Pergamon 2001:14794-800.
32. Ministry of Health. The Ghana health sector 2008 programme of work. Accra: GoG, 2007.
[dataset]33. Bondarenko M., Kerr D., Sorichetta A., et al. Estimates of total number of people per grid square, adjusted to match the corresponding UNPD 2020 estimates and broken down by gender and age groupings, produced using Ecopia.AI and Maxar Technologies building footprints. [dataset]. 2020 doi: 10.5258/SOTON/WP00699
34. Kobo Inc. KoBotoolbox [Computer Sofware Application] 2022 [Available from: https://www.kobotoolbox.org/kobo/.
35. Greco S, Ishizaka A, Tasiou M, et al. On the Methodological Framework of Composite Indices: A Review of the Issues of Weighting, Aggregation, and Robustness. *Social Indicators Research* 2019;141(1):61-94. doi: 10.1007/s11205-017-1832-9
36. Countdown. Mind the gap: equity and trends in coverage of maternal, newborn, and child health services in 54 Countdown countries. *The Lancet* 2008;371(9620):1259-67. doi: 10.1016/S0140-6736(08)60560-7

For peer review only

37. Escamilla V, Calhoun L, Winston J, et al. The Role of Distance and Quality on Facility Selection for Maternal and Child Health Services in Urban Kenya. *Journal of Urban Health* 2018;95(1):1-12. doi: 10.1007/s11524-017-0212-8
[dataset]38. OpenStreetMap Contributors. Open geospatial map data [available online] 2022 [Available from: https://www.openstreetmap.org/ accessed 12/03/2022.
[dataset]39. Zanaga D, Van De Kerchove R, De Keersmaecker W, et al. ESA WorldCover 10 m 2020 v100 [dataset]. 2021 doi: https://doi.org/10.5281/zenodo.5571936
40. Ray N, Ebener S. AccessMod 3.0: computing geographic coverage and accessibility to health care services using anisotropic movement of patients. *International Journal of Health Geographics* 2008;7(1):63. doi: 10.1186/1476-072X-7-63
41. Alegana VA, Wright JA, Pentrina U, et al. Spatial modelling of healthcare utilisation for treatment of fever in Namibia. *International Journal of Health Geographics* 2012;11(1):6. doi: 10.1186/1476-072X-11-6
42. Tobler W. Three presentations on geographical analysis and modeling: National Center for Geographic Information and Analysis. *Santa Barbara, CA93106-4060: University of California, Santa Barbara* 1993
43. Farr TG, Rosen PA, Caro E, et al. The Shuttle Radar Topography Mission. *Reviews of Geophysics* 2007;45(2) doi: https://doi.org/10.1029/2005RG000183
[dataset]44. (USGS) UGS. Shuttle Radar Topography Mission 1 Arc-Second Global [dataset]. 2000 doi: 10.5066/F7F76B1X
45. WHO, Unicef. Monitoring emergency obstetric care: a handbook: Geneva: World Health Organization, 2009.
46. Wilson AG. A family of spatial interaction models, and associated developments. *Environment and Planning A* 1971;3(1):1-32.
47. Tegegne TK, Chojenta C, Loxton D, et al. The impact of geographic access on institutional delivery care use in low and middle-income countries: Systematic review and meta-analysis. *PLOS ONE* 2018;13(8):e0203130. doi: 10.1371/journal.pone.0203130
48. Burger M, van Oort F, Linders G-J. On the Specification of the Gravity Model of Trade: Zeros, Excess Zeros and Zero-inflated Estimation. *Spatial Economic Analysis* 2009;4(2):167-90. doi: 10.1080/17421770902834327
49. Congdon P. Approaches to Modelling Overdispersion in the Analysis of Migration. *Environment and Planning A: Economy and Space* 1993;25(10):1481-510. doi: 10.1068/a251481
50. Fotheringham AS, O'Kelly ME. Spatial interaction models: formulations and applications. Dordrecht, The Netherlands: Kluwer Academic Publishers Dordrecht 1989.
51. Burnham KP, Anderson DR. Model selection and multimodel inference: A practical information-theoretic approach. 2nd ed: Springer New York 2002.
52. Gabrysch S, Cousens S, Cox J, et al. The Influence of Distance and Level of Care on Delivery Place in Rural Zambia: A Study of Linked National Data in a Geographic Information System. *PLOS Medicine* 2011;8(1):e1000394. doi: 10.1371/journal.pmed.1000394
53. Kruk ME, Leslie HH, Verguet S, et al. Quality of basic maternal care functions in health facilities of five African countries: an analysis of national health system surveys. *The Lancet Global Health* 2016;4(11):e845-e55. doi: 10.1016/S2214-109X(16)30180-2
54. Sumankuuro J, Crockett J, Wang S. Perceived barriers to maternal and newborn health services delivery: a qualitative study of health workers and community

For peer review only

members in low and middle-income settings. *BMJ Open* 2018;8(11):e021223. doi: 10.1136/bmjopen-2017-021223
55. Afaya A, Dzomeku VM, Baku EA, et al. Women's experiences of midwifery care immediately before and after caesarean section deliveries at a public Hospital in the Western Region of Ghana. *BMC Pregnancy and Childbirth* 2020;20(1):8. doi: 10.1186/s12884-019-2698-4
56. Gabrysch S, Civitelli G, Edmond KM, et al. New Signal Functions to Measure the Ability of Health Facilities to Provide Routine and Emergency Newborn Care. *PLOS Medicine* 2012;9(11):e1001340. doi: 10.1371/journal.pmed.1001340
57. Dettrick Z, Firth S, Jimenez Soto E. Do Strategies to Improve Quality of Maternal and Child Health Care in Lower and Middle Income Countries Lead to Improved Outcomes? A Review of the Evidence. *PLOS ONE* 2013;8(12):e83070. doi: 10.1371/journal.pone.0083070
58. Adatara P, Strumpher J, Ricks E. Exploring the reasons why women prefer to give birth at home in rural northern Ghana: a qualitative study. *BMC Pregnancy and Childbirth* 2020;20(1):500. doi: 10.1186/s12884-020-03198-y
59. Wong KLM, Benova L, Campbell OMR. A look back on how far to walk: Systematic review and meta-analysis of physical access to skilled care for childbirth in Sub-Saharan Africa. *PLOS ONE* 2017;12(9):e0184432. doi: 10.1371/journal.pone.0184432
60. Bailey P, Lobis S, Maine D, et al. Monitoring emergency obstetric care: a handbook: World Health Organization 2009.
61. Say L, Chou D, Gemmill A, et al. Global causes of maternal death: a WHO systematic analysis. *The Lancet Global Health* 2014;2(6):e323-e33. doi: https://doi.org/10.1016/S2214-109X(14)70227-X
62. Sriram V, Bennett S. Strengthening medical specialisation policy in low-income and middle-income countries. *BMJ Global Health* 2020;5(2):e002053. doi: 10.1136/bmjgh-2019-002053
63. GHS. National assessment for emergency obstetric and newborn care, Ghana, 2011.
64. van Duinen AJ, Adde HA, Fredin O, et al. Travel time and perinatal mortality after emergency caesarean sections: an evaluation of the 2-hour proximity indicator in Sierra Leone. *BMJ Global Health* 2020;5(12):e003943. doi: 10.1136/bmjgh-2020-003943
65. Stewart Fotheringham A, Rogerson PA. GIS and spatial analytical problems. *International Journal of Geographical Information Systems* 1993;7(1):3-19. doi: 10.1080/02693799308901936
66. Pezzulo C, Hornby GM, Sorichetta A, et al. Sub-national mapping of population pyramids and dependency ratios in Africa and Asia. *Scientific Data* 2017;4(1):170089. doi: 10.1038/sdata.2017.89
67. Fries B, Guerra CA, García GA, et al. Measuring the accuracy of gridded human population density surfaces: A case study in Bioko Island, Equatorial Guinea. *PLOS ONE* 2021;16(9):e0248646. doi: 10.1371/journal.pone.0248646

Supplementary material

Supplemental appendix 1: Comparison between demand aggregated at sub-district versus travel time to place of residence cost allocation

Supplemental appendix 2: Travel speed assigned to land cover and road types

Supplemental appendix 3: Proportionate weighted estimation of mechanised travel speeds by rood smoothness and road class

Supplemental appendix 4:Relative performance of quality care indices in predicting the count of women giving birth between town-health facilities

Supplemental appendix 5: Breakdown of data availability and missing data by health facility ownership

Supplemental appendix 6: Correlation between (A) Aggregated versus individual data, (B) Individual data versus geocoded individual data, and (C) Aggregated versus individual geocoded data

For peer review only

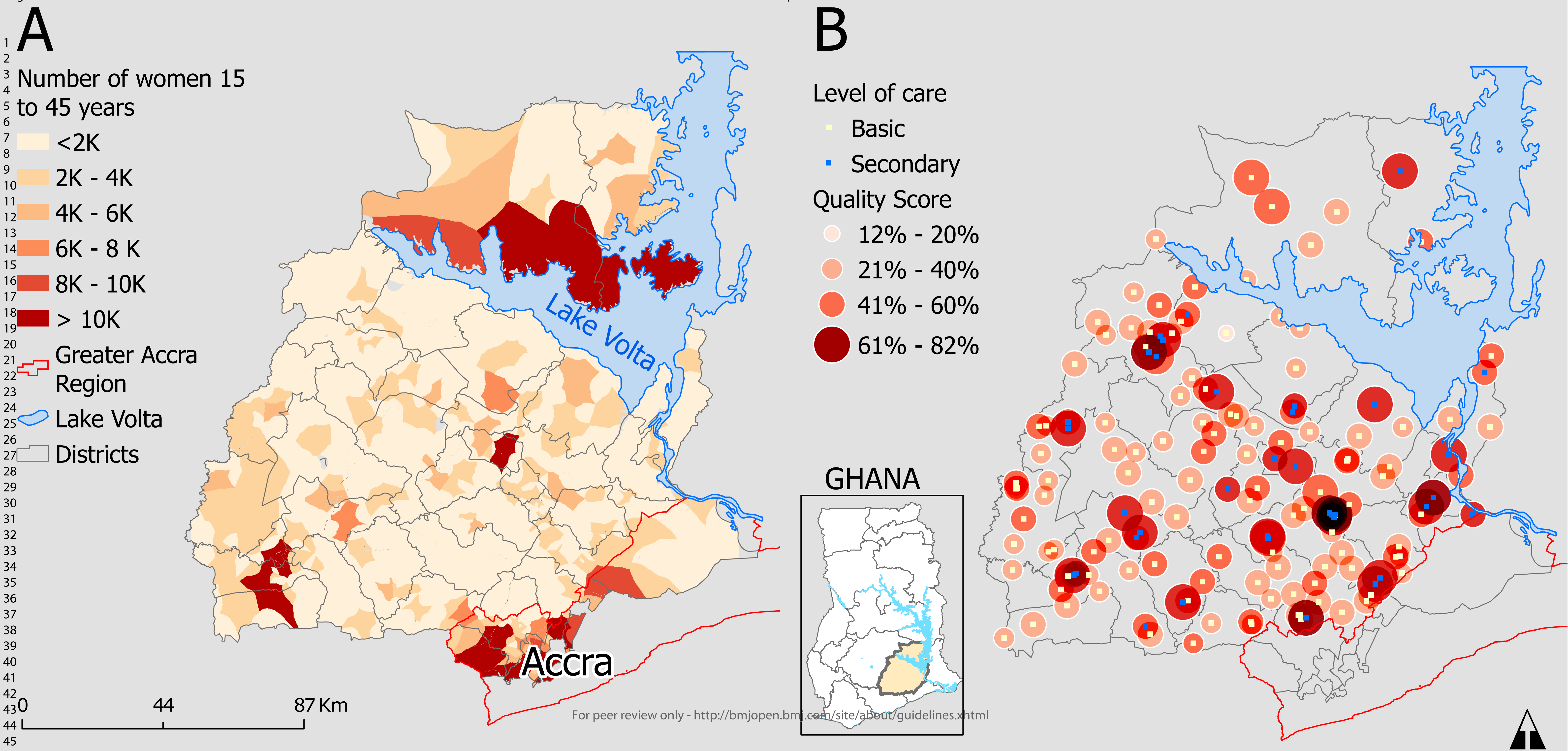

For peer review only - http://bmjopen.bmj.com/site/about/guidelines.xhtml

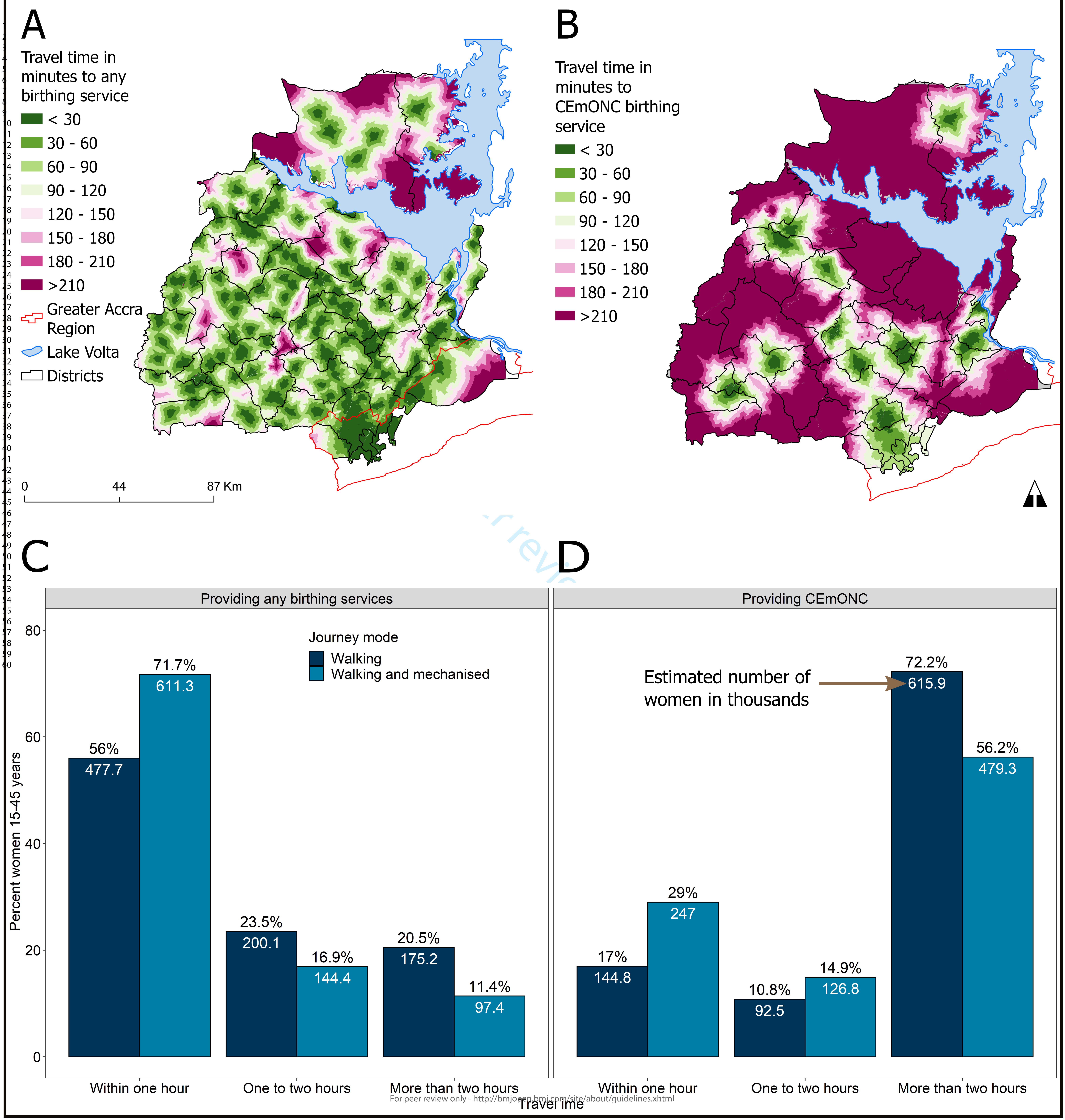
A
Travel time in minutes to any birthing service
< 30
30 - 60
60 - 90
90 - 120
120 - 150
150 - 180
180 - 210
>210
Greater Accra Region
Lake Volta
Districts
0
44
87 Km
B
Travel time in minutes to CEmONC birthing service
< 30
30 - 60
60 - 90
90 - 120
120 - 150
150 - 180
180 - 210
>210
C
Providing any birthing services
Journey mode
Walking
Walking and mechanised
71.7%
611.3
56%
477.7
23.5%
200.1
16.9%
144.4
20.5%
175.2
11.4%
97.4
D
Providing CEmONC
Estimated number of women in thousands
72.2%
615.9
56.2%
479.3
29%
247
17%
144.8
10.8%
92.5
14.9%
126.8
Percent women 15-45 years
80
60
40
20
0
Within one hour
One to two hours
More than two hours
Travel ime

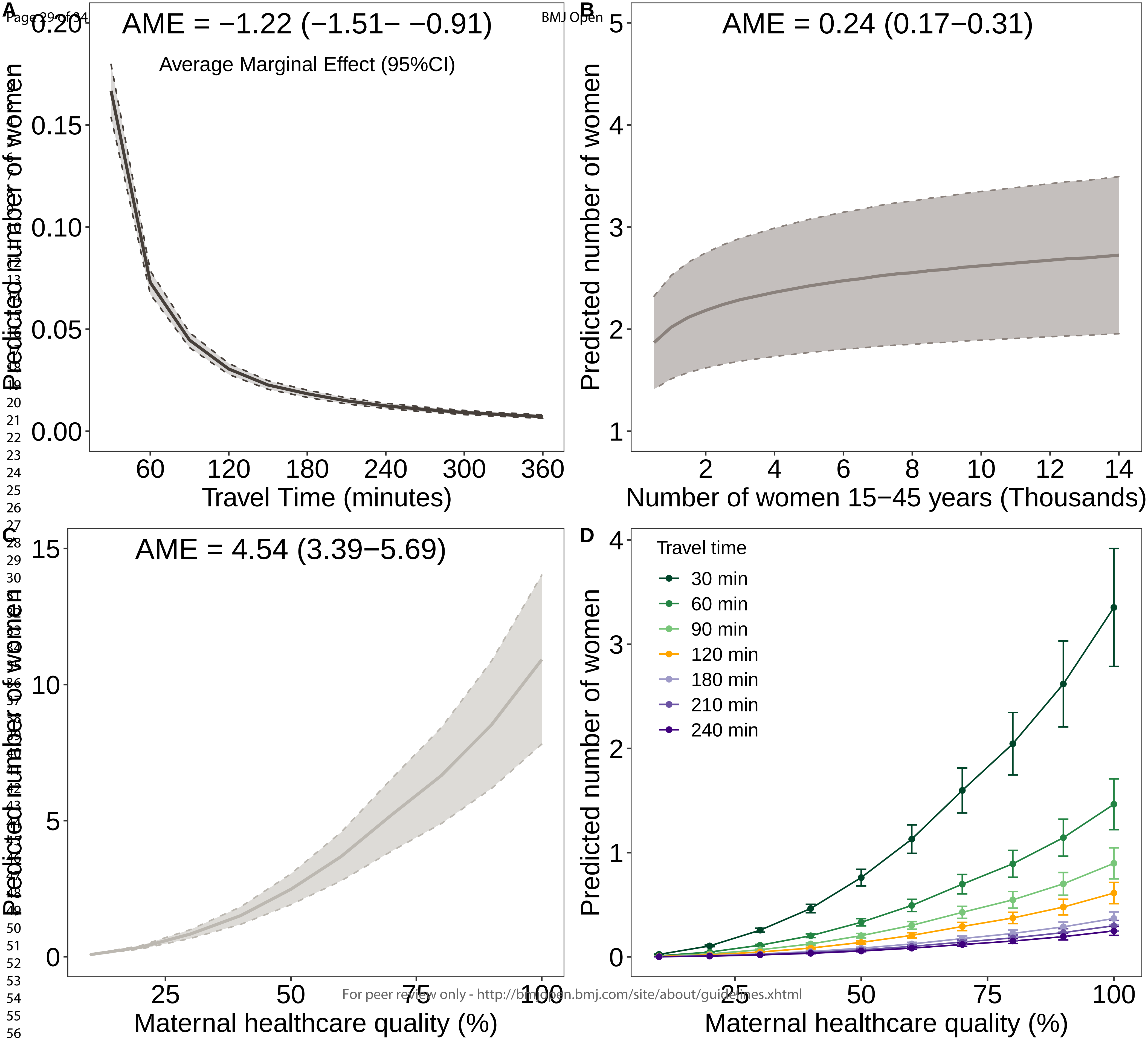
A
AME = −1.22 (−1.51− −0.91)
Average Marginal Effect (95%CI)
Predicted number of women
0.00
0.05
0.10
0.15
0.20
60
120
180
240
300
360
Travel Time (minutes)
B
AME = 0.24 (0.17−0.31)
Predicted number of women
1
2
3
4
5
2
4
6
8
10
12
14
Number of women 15−45 years (Thousands)
C
AME = 4.54 (3.39−5.69)
Predicted number of women
0
5
10
15
25
50
75
100
Maternal healthcare quality (%)
D
Travel time
30 min
60 min
90 min
120 min
180 min
210 min
240 min
Predicted number of women
0
1
2
3
4
25
50
75
100
Maternal healthcare quality (%)

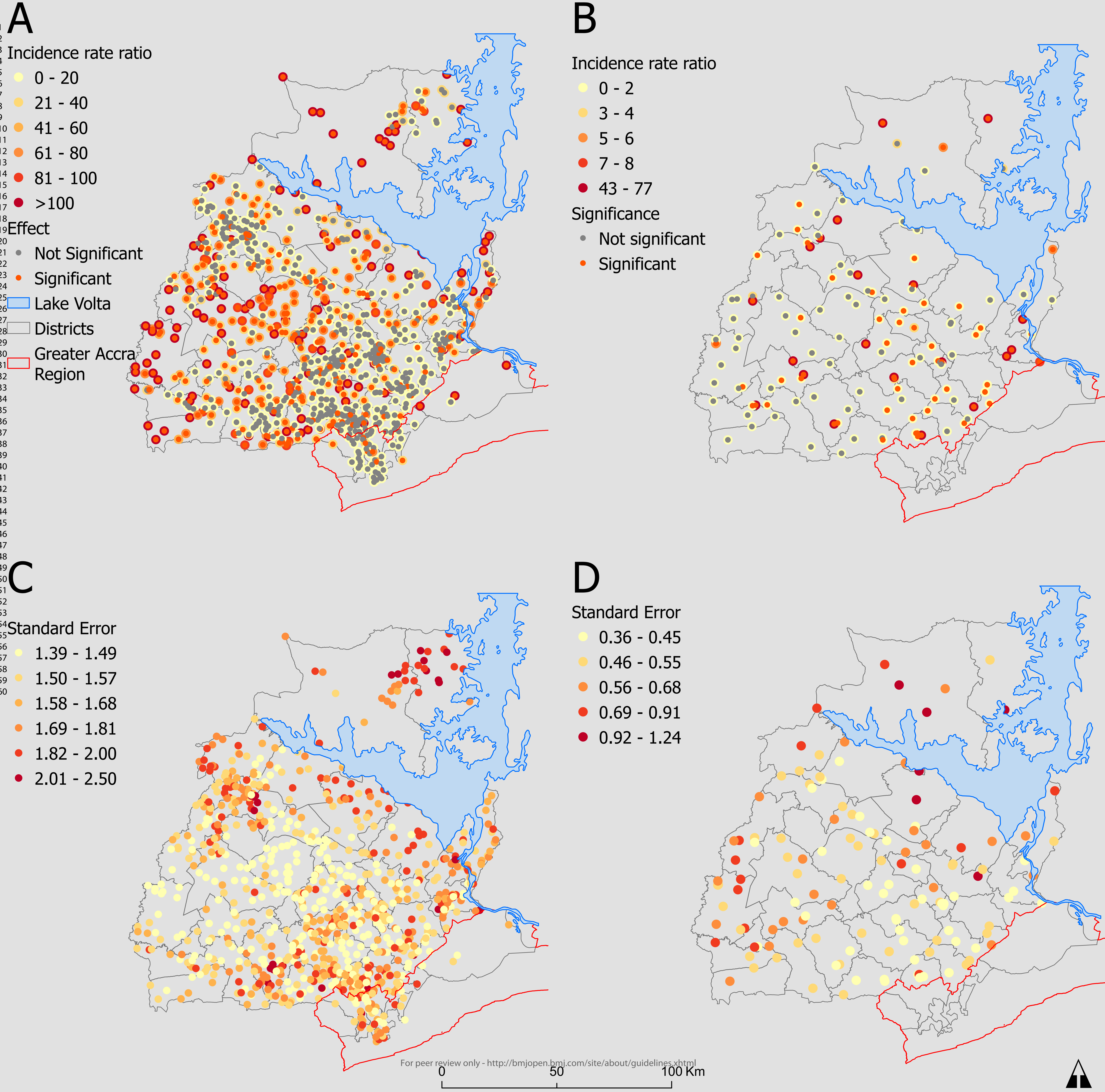
A
Incidence rate ratio
0 - 20
21 - 40
41 - 60
61 - 80
81 - 100
>100
Effect
Not Significant
Significant
Lake Volta
Districts
Greater Accra Region
B
Incidence rate ratio
0 - 2
3 - 4
5 - 6
7 - 8
43 - 77
Significance
Not significant
Significant
C
Standard Error
1.39 - 1.49
1.50 - 1.57
1.58 - 1.68
1.69 - 1.81
1.82 - 2.00
2.01 - 2.50
D
Standard Error
0.36 - 0.45
0.46 - 0.55
0.56 - 0.68
0.69 - 0.91
0.92 - 1.24
0
50
100 Km

Supplementary material

Supplemental appendix 1: Comparison between demand aggregated at sub-district versus travel time to place of residence cost allocation

Model	AIC	AIC Change
Travel time + summary quality index + number of women 15 to 45 years near the catchment of a town (origin) estimated by travel time to place of residence cost allocation.	84,796.6	0
Travel time + summary quality index + number of women 15 to 45 years in origin sub-district	92,298.1	7501.47

Supplemental appendix 2: Travel speed assigned to land cover and road types

Feature	Travel speeds (Kmh^{-1})	
	Multi modal	Walking
Land cover		
Grassland and built-up areas	5	5
Trees, shrubland, cropland, bare or sparse vegetation	4	4
Herbaceous wetland	3	3
Rivers and lakes	0	0
Roads		
Trunk	48	5
Primary	43	5
Secondary	40	5
Tertiary	35	5
Tracks and residential	30	5
Footpaths	5	5

Supplemental appendix 3: Proportionate weighted estimation of mechanised travel speeds by rood smoothness and road class

Road smoothness description by OSM	Average speed by road smoothness (A)	% primary (B)	A*B	% secondary (C)	A*C	% tertiary (D)	A*D	% track (E)	A*E	% trunk (F)	A*F	% All roads (G)	A*G
Horrible	20	0	0.0	2	0.3	1	0.2	9	1.8	0	0.0	4	0.7
Very bad	31	14	4.2	16	4.8	58	18.0	80	24.9	1	0.3	45	14.0
bad	31	16	5.1	30	9.2	19	5.9	9	2.9	1	0.3	14	4.2
good	48	65	31.0	49	23.6	16	7.6	1	0.4	98	46.9	35	16.7
intermediate	48	5	2.6	4	1.9	5	2.4	0	0.1	0	0.0	2	1.2
excellent	48	0	0.0	0	0.0	1	0.4	0	0.1	0	0.0	0	0.1
Speed applied to road types		100	43	100	40	100	35	100	30	100	48	100	37

For peer review only

The OSM road smoothness are described here:
https://wiki.openstreetmap.org/wiki/Key:smoothness

Supplemental appendix 4:Relative performance of quality care indices in predicting the count of women giving birth between town-health facilities

**Model**	**AIC**	**AIC Change**
Travel time + number of women 15 to 45 years in origin sub-district + summary quality index (human resource capacity, signal functions, medicines, logistics, amenities, referral capacity, motivation, privacy, training and wash)	84,796.65	0
Travel time + number of women 15 to 45 years in origin sub-district + summary quality index quintiles	84,885.28	88.54
Travel time + number of women 15 to 45 years in origin sub-district + size of health facility (human resource capacity, signal functions, medicines, logistics, amenities, referral capacity)	84,328.42	502.76
Travel time + number of women 15 to 45 years in origin sub-district +health facility type (CHPS, health center, hospital, polyclinic, maternity home)	85,407.18	610.53
Travel time + number of women 15 to 45 years in origin sub-district + Number of EmONC signal functions	85,953.44	1156.79
Travel time + number of women 15 to 45 years in origin sub-district +health facility level (primary or secondary)	86,078.33	1281.68
Travel time + number of women 15 to 45 years in origin sub-district + routine quality (staff motivation, privacy, training, wash)	89,608.96	5478.0.03

For peer review only

Supplemental appendix 5: Breakdown of data availability and missing data by health facility ownership

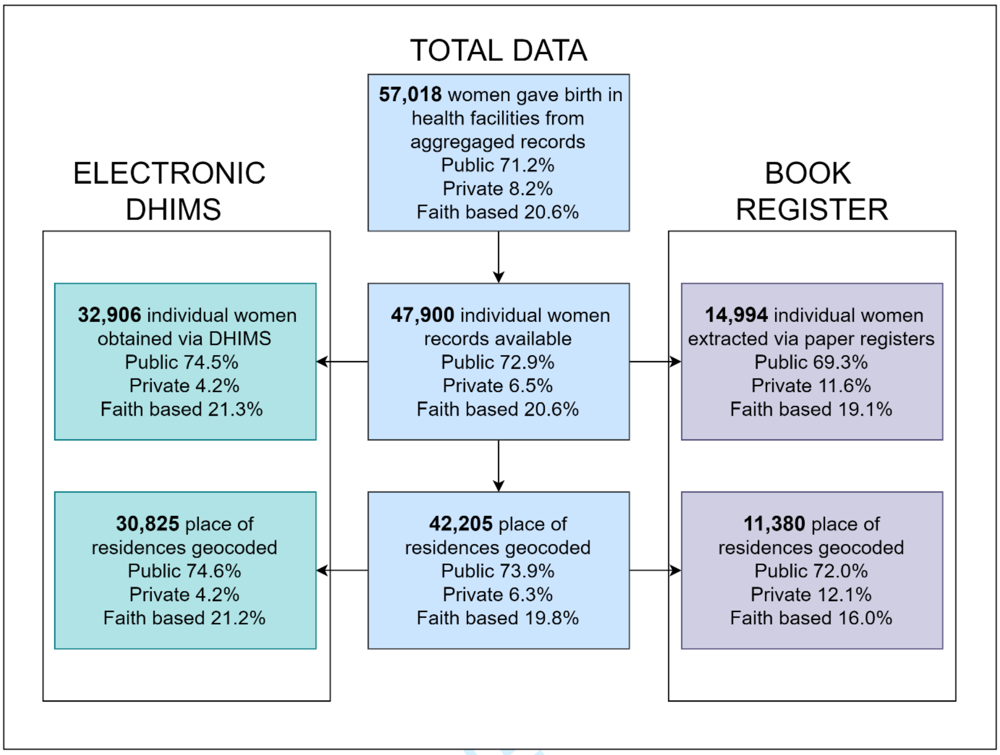

Supplemental appendix 6: Correlation between (A) Aggregated versus individual data, (B) Individual data versus geocoded individual data, and (C) Aggregated versus individual geocoded data

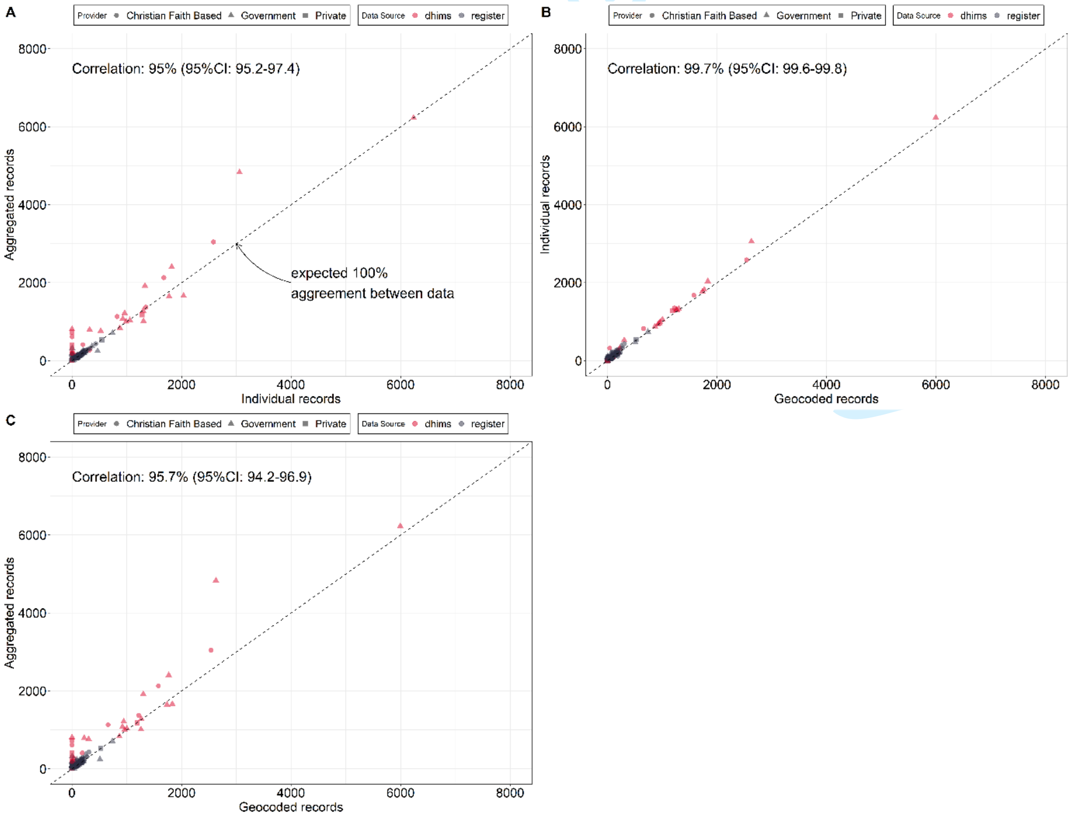

STROBE Statement—Checklist of items that should be included in reports of ***cross-sectional studies***

	Item No	Recommendation	Lines
**Title and abstract**	1	(*a*) Indicate the study's design with a commonly used term in the title or the abstract	1 – 3
		(*b*) Provide in the abstract an informative and balanced summary of what was done and what was found	33 - 52
**Introduction**			
Background/rationale	2	Explain the scientific background and rationale for the investigation being reported	82 – 130
Objectives	3	State specific objectives, including any prespecified hypotheses	131 - 139
**Methods**			
Study design	4	Present key elements of study design early in the paper	141 - 145
Setting	5	Describe the setting, locations, and relevant dates, including periods of recruitment, exposure, follow-up, and data collection	147 – 155
Participants	6	(*a*) Give the eligibility criteria, and the sources and methods of selection of participants	156 - 170
Variables	7	Clearly define all outcomes, exposures, predictors, potential confounders, and effect modifiers. Give diagnostic criteria, if applicable	234 - 271
Data sources/ measurement	8*	For each variable of interest, give sources of data and details of methods of assessment (measurement). Describe comparability of assessment methods if there is more than one group	*157-159, 172 – 173, 185 – 190, 210 – 218, 255 - 271*
Bias	9	Describe any efforts to address potential sources of bias	488 - 506
Study size	10	Explain how the study size was arrived at	169 - 170<br>182 - 186
Quantitative variables	11	Explain how quantitative variables were handled in the analyses. If applicable, describe which groupings were chosen and why	157 - 170<br>255 - 271
Statistical methods	12	(*a*) Describe all statistical methods, including those used to control for confounding	184 - 271
		(*b*) Describe any methods used to examine subgroups and interactions	265 - 267
		(*c*) Explain how missing data were addressed	157 – 159<br>188 - 190
		(*d*) If applicable, describe analytical methods taking account of sampling strategy	234 - 271
		(*e*) Describe any sensitivity analyses	-
**Results**			
Participants	13*	(a) Report numbers of individuals at each stage of study—eg numbers potentially eligible, examined for eligibility, confirmed eligible, included in the study, completing follow-up, and analysed	285 – 289
		(b) Give reasons for non-participation at each stage	285 - 289<br>Supplemental material
		(c) Consider use of a flow diagram	Supplemental

For peer review only - http://bmjopen.bmj.com/site/about/guidelines.xhtml

			material
Descriptive data	14*	(a) Give characteristics of study participants (eg demographic, clinical, social) and information on exposures and potential confounders	
		(b) Indicate number of participants with missing data for each variable of interest	285 - 289 Supplemental material
Outcome data	15*	Report numbers of outcome events or summary measures	285 – 289 314 - 315
Main results	16	(*a*) Give unadjusted estimates and, if applicable, confounder-adjusted estimates and their precision (eg, 95% confidence interval). Make clear which confounders were adjusted for and why they were included	328 - 367
		(*b*) Report category boundaries when continuous variables were categorized	-
		(*c*) If relevant, consider translating estimates of relative risk into absolute risk for a meaningful time period	-
Other analyses	17	Report other analyses done—eg analyses of subgroups and interactions, and sensitivity analyses	361 – 369
**Discussion**			
Key results	18	Summarise key results with reference to study objectives	391 – 398
Limitations	19	Discuss limitations of the study, taking into account sources of potential bias or imprecision. Discuss both direction and magnitude of any potential bias	489 – 523
Interpretation	20	Give a cautious overall interpretation of results considering objectives, limitations, multiplicity of analyses, results from similar studies, and other relevant evidence	399 – 478
Generalisability	21	Discuss the generalisability (external validity) of the study results	479 - 488
**Other information**			
Funding	22	Give the source of funding and the role of the funders for the present study and, if applicable, for the original study on which the present article is based	560 - 564

*Give information separately for exposed and unexposed groups.

Note: An Explanation and Elaboration article discusses each checklist item and gives methodological background and published examples of transparent reporting. The STROBE checklist is best used in conjunction with this article (freely available on the Web sites of PLoS Medicine at http://www.plosmedicine.org/, Annals of Internal Medicine at http://www.annals.org/, and Epidemiology at http://www.epidem.com/). Information on the STROBE Initiative is available at www.strobe-statement.org.

For peer review only
